# Recent advances in peptide-based therapeutic strategies for breast cancer treatment

**DOI:** 10.3389/fphar.2023.1052301

**Published:** 2023-01-30

**Authors:** Ling Li, Gregory J. Duns, Wubliker Dessie, Zhenmin Cao, Xiaoyuan Ji, Xiaofang Luo

**Affiliations:** ^1^ Hunan Engineering Technology Research Center for Comprehensive Development and Utilization of Biomass Resources, College of Chemistry and Bioengineering, Hunan University of Science and Engineering, Yongzhou, China; ^2^ Academy of Medical Engineering and Translational Medicine, Medical College, Tianjin University, Tianjin, China

**Keywords:** peptides, breast cancer, targeted vector, CPP, Cancer Vaccines, anticancer drugs

## Abstract

Breast cancer is the leading cause of cancer-related fatalities in female worldwide. Effective therapies with low side effects for breast cancer treatment and prevention are, accordingly, urgently required. Targeting anticancer materials, breast cancer vaccines and anticancer drugs have been studied for many years to decrease side effects, prevent breast cancer and suppress tumors, respectively. There are abundant evidences to demonstrate that peptide-based therapeutic strategies, coupling of good safety and adaptive functionalities are promising for breast cancer therapy. In recent years, peptide-based vectors have been paid attention in targeting breast cancer due to their specific binding to corresponding receptors overexpressed in cell. To overcome the low internalization, cell penetrating peptides (CPPs) could be selected to increase the penetration due to the electrostatic and hydrophobic interactions between CPPs and cell membranes. Peptide-based vaccines are at the forefront of medical development and presently, 13 types of main peptide vaccines for breast cancer are being studied on phase III, phase II, phase I/II and phase I clinical trials. In addition, peptide-based vaccines including delivery vectors and adjuvants have been implemented. Many peptides have recently been used in clinical treatments for breast cancer. These peptides show different anticancer mechanisms and some novel peptides could reverse the resistance of breast cancer to susceptibility. In this review, we will focus on current studies of peptide-based targeting vectors, CPPs, peptide-based vaccines and anticancer peptides for breast cancer therapy and prevention.

## 1 Introduction

Cancer is a common cause of death, especially in the developing countries (Dissanayake et al., 2017). It resulted in over 9.95 million human deaths in 2020 provided by GLOBOCAN. Among female cancer patients, breast, lung, colorectal, cervical, and gastric cancers are the most common types of cancer. In these cancers, breast cancer is the second major cause of cancer-related fatalities worldwide in female (DeSantis et al., 2014) and has been the commonest cancer diagnosed in clinic until December 2020, according to statistics released by the International Agency for Research on Cancer (IARC). According to the data reported by Siegel et al., there were approximately 279,110 breast cancer patients in women and men, and about 48,530 *in situ* breast cancer cases in the United States alone (Siegel et al., 2020). The average 5-year survival rate of progressed metastatic breast cancer patients is only 22% (Terjung et al., 2014). However, the 5-year survival rate of patients with breast cancer in China has a gap of approximately 8% compared with those of the United States and Europe (Ma et al., 2021). Based on the estrogen receptor (ER), progesterone receptor and human epidermal growth factor receptor-2 (HER2) status, there are three breast cancer subtypes: HER2-positive, hormone receptor-positive and triple negative subtypes (Parise and Caggiano, 2014). Among all the breast cancer, hormone receptor-positive breast cancer constitutes approximately 60%–70% (Hart et al., 2015), while the proportion of triple negative breast cancer (TNBC) is about 15%–20% (Marra et al., 2019), and the remainder are HER2-positive breast cancer subtype. Although breast cancer is a major cause of cancer-related fatalities in female (Loftus et al., 2021), it may well be cure if it is early diagnosed and appropriately treated.

There are several traditional treatments for breast cancer: targeted therapy (Gilbert et al., 2021), radiotherapy, chemotherapy, surgery (Riis, 2020), and hormone therapy, often together or in various combinations which depend on the breast cancer subtype. Among these treatments (Blanco et al., 2011; Hou et al., 2019), chemotherapy plays a basic role for treating breast cancer in clinic (Pérez-Herrero and Fernandez-Madarde, 2015; Parikh et al., 2017) and will keep up integration as a backbone in the treatment of breast cancer for the near-future (Maeda, 2015; Claessens et al., 2020). However, the high dosage of chemotherapy drugs ultimately leads to serious side effects and multi-drug resistance (Fan et al., 2016; Suo et al., 2016). Thus, the goal is to reduce or even avoid toxic side effects and resistance (Suo et al., 2017).

**FIGURE 1:**
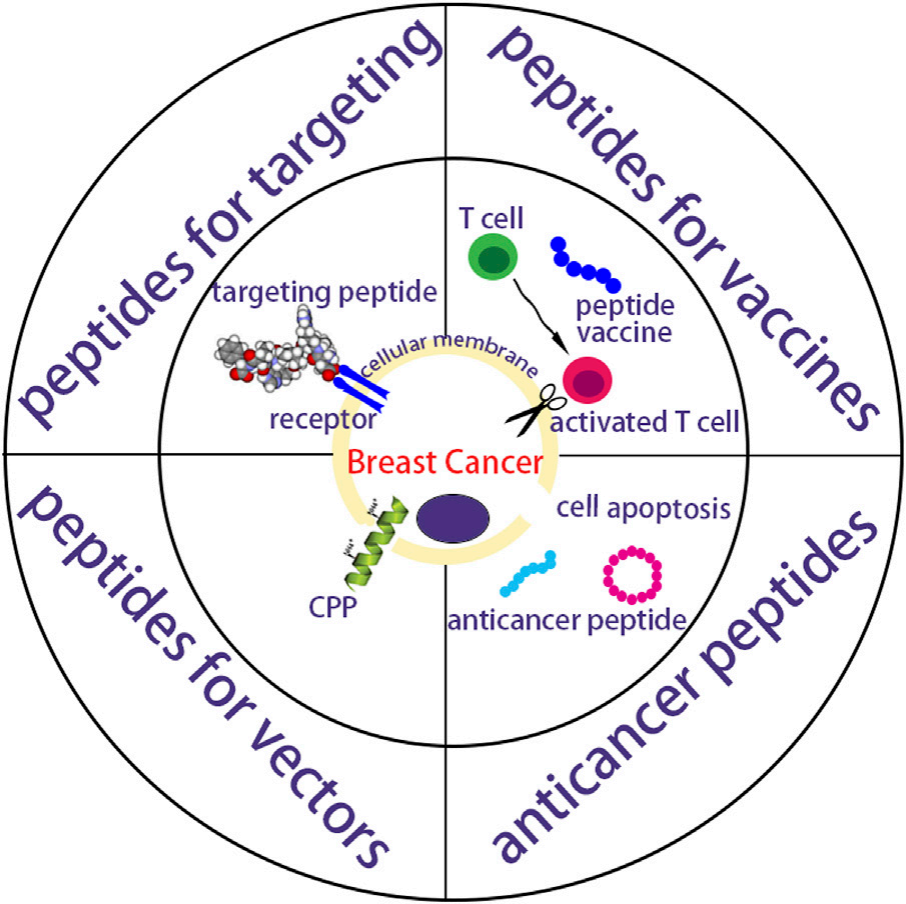
A schematic summarization of peptides as targeting vectors, vaccines and anticancer drugs for breast cancer.

Peptides exhibit scalable production, good safety and adaptive functionalities, and are important for delivering different anticancer drugs like biopharmaceutics and chemical drugs (Deming, 2016; Mazo et al., 2020). The drug-free neutrally charged peptide, containing a cationic peptide guanidinium-functionalized poly(L-lysine) and a pH-sensitive peptide poly(ethylene glycol)-b-poly(ʟ-lysine)-graft-cyclohexene-1,2-dicarboxylic anhydride, exhibited similar efficacy against resistant MCF-7/ADR cells and MCF-7 cells *in vitro* (Yang et al., 2022). These neutrally charged peptides also circumvented *in vivo* instability and toxicity of cationic charged anticancer medicines. The phenylalanine dipeptide (FF) can self-assemble by π-π stacking (Reches and Gazit, 2003) into either vesicular structures or a nanotube structure at low concentrations (<7 mg/mL) or higher concentration (>10 mg/mL) respectively (Song et al., 2004). Wang et al. synthesized RAFF modified with polyethylene glycol (PEG-RAFF) self-assembled nanospheres for surviving siRNA delivery (Han et al., 2019). In the HER2 extracellular domain, antigenic peptide CH401 (YQDTILWKDIFHKNNQLALT) was expected to effectively activate both CD4^+^ and CD8^+^ and thus induce T cell response (Ishida et al., 1994; Miyako et al., 2011), and co-assembling vaccines composed of peptide CH401 and additional lipid chains had enhanced vaccine potency (Aiga et al., 2020). In this review, peptides working as targeted nano-vectors, cancer vaccines and anticancer drugs in treating breast cancer are highlights (Figure 1). Anticancer strategies involving peptides will show increasing clinical translation in the future.

## 2 Peptides for anticancer drug vectors

Nucleic acid therapy appears to be a promising strategy of cancer treatment for its abilities to overcome many challenges such as metastasis, drug resistance, genetic alterations, tumor microenvironment heterogeneity, and tumor relapse (Jin et al., 2020). However, nucleic acid therapy containing small interfering RNA (He et al., 2014), microRNA, aptamers (Zhou et al., 2016), and immune adjuvant nucleic acids (Zhang et al., 2018), still faces several obstacles: low solubility, instability, weak permeability, uncontrolled drug release kinetics and lack of target specificity. Some organic materials including liposomes (Eckstein, 2014), polymersomes, micelles, peptides and dendrimers work as nanotechnology vectors for protecting nucleic acids and targeting tumor cells (Chan et al., 2009; Pan et al., 2017). Among these targeting vectors, peptides (Shukla et al., 2014; Numata, 2015) have attracted a lot of attention due to the ease of manipulation and inherent biocompatibility (Zhao and Jiang, 2018; Liu et al., 2019). In addition to good biocompatibility (Singh et al., 2018; Cummings et al., 2019), peptides (Tai and Gao, 2017; Yang et al., 2018) with desirable sequences (Wang et al., 2018; Zhang and Lu, 2018) could further enhance the transfection efficacy (Li et al., 2018; Kuai et al., 2019).

### 2.1 Peptides for targeting breast cancer

**FIGURE 2:**
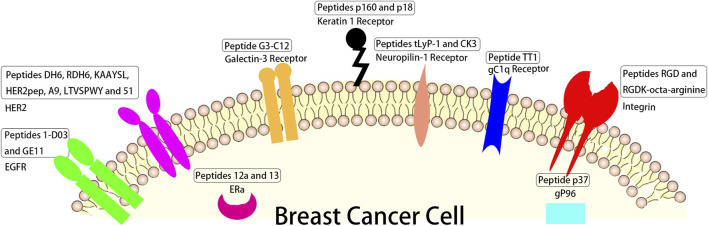
The schematic of different peptides targeting breast tumor and their corresponding targets overexpressed in breast tumor cells.

Heat shock protein (HSP) gp96 (Hou et al., 2015), a molecular chaperone residing in the cell membrane of cancer cells, is usually used as a target in cancer treatment (Hou et al., 2015; Li et al., 2015). Peptide p37 (LNVSRETLQQHKLLKVIRKKLVRKTLDMIKKIADDKY), which can specifically recognize the N-terminal helix-loop-helix sequence of gp96, is able to disrupt intramolecular helix-helix interaction and inhibit gp96 conformational changes, thereby destroying its chaperoning function (Li et al., 2015; Qian et al., 2019). Liang et al. (Liang et al., 2021) modified cationic liposome CDO14 with p37 to form p37-CDO14 with gp96-targeted function and found that p37-CDO14 could specifically bind to gp96-overexpressed breast cancer cells on cytomembrane (Figure 2).

3-mer peptide RGD prepared by Pierschbacher and Ruoslahti from fibronectin (Pierschbacher and Ruoslahti, 1984; Garate et al., 2015), can recognize several integrins, some glycoproteins composed of α and β subunits (Demircioglu and Hodivala-Dike, 2016; Cheng and Ji, 2019) which are overexpressed on the cancer cell membrane and involved in angiogenesis, tumor growth (Nieberler et al., 2017) and metastasis (Wu et al., 2019; Chen et al., 2021). Hazeri et al. (Hazeri et al., 2022) fabricated a targeting delivery system for doxorubicin (DOX) with mesoporous silica nanoparticles loaded with AS1411 aptamer and peptide RGDK-octa-arginine. The results revealed this targeting delivery system could obviously enhance the anticancer activity of DOX with single-dose administration *in vivo* (Figure 2).

Linear peptide TT1 (AKRGARSTA) was often used to modify the surface of liposomes for targeting protein p32 (Simón-Gracia et al., 2018; Simón-Gracia et al., 2018), known as a transmembrane gC1q receptor, which is overexpressed on the cellular surface of cancer and cancer associated cells, like active angiogenic endothelial cells (Rubinstein et al., 2004), cancer associated fibroblast (Fogal et al., 2008), and cancer associated macrophages (TAMs) (Agemy et al., 2013; Sharma et al., 2017). The cell results showed that the liposome modified with linear peptide TT1 had better interaction with 3D breast cancer spheroids than non-modified one and that 50% of the total nanovesicles were internalized in M2 primary human macrophages (d’Avanzo et al., 2021) (Figure 2).

Peptide tLyP-1 (CGNKRTR), a tumor homing ligand, can specifically target neuropilin-1 receptor overexpressed in breast cancer (Bielenberg et al., 2006; Zhu et al., 2018). Zhong et al. found that tLyP-1 peptide-functionalized chimaeric polymersomes showed well ability of loading of siNAC-1 (up to 14.4 wt. %) and potently inhibited MDA-MB-231 cell invasion and migration (Wang et al., 2020a). Peptide CK3 (CLKADKAKC) as described by Feng et al. (Feng et al., 2014) showed higher ability binding to MDA-MB-231 breast tumor cells than other breast tumor cells such as 4T-1, MCF-7 and MDA-MB-435 breast tumor cells (Figure 2).

Linear dodecapeptide p160 (VPWMEPAYQRFL) displays strong binding to MCF-7 and MDA-MB-435 breast tumor cells according to the target of keratin 1 receptor (Askoxylakis et al., 2005). Its analogue peptide p18 (WXEAAYQRFL), proteolytically stable in human serum, binds to keratin 1 receptor (Shahin et al., 2013), and DOX loaded P18-PEtOx-dioleoyl phosphatidyl ethanolamine (DOPE) nanoliposomes designed for targeting AU565 cells elicited a strong antitumor response compared to free DOX (Bolat et al., 2021) (Figure 2).

Peptide G3-C12 (ANTPCGPYTHDCPVKR) specific binding to galectin-3 receptor, which is a 30 kDa protein and associated with cancer metastases and growth in breast cancer (Morris et al., 2004), was connected with 1,4,7,10-tetra-azacyclododecane-N,N′,N″N‴-tetraacetic acid (DOTA) by a peptide GSG linker which was radiolabeled with 111In for breast cancer targeting (Kumar and Deutscher, 2008). The results showed the peptide G3-C12 can significantly bind to human MDA-MB-435 (galectin-3) cells (Figure 2).

ERs contain ERα and ERβ (Nilsson et al., 2011), in which ERα activated for proliferation of breast and uterine tissues (Frasor et al., 2003; Helguero et al., 2005) was overexpressed in more than 70% of breast cancers (Holst et al., 2007), thus ERα is the an important target for treating ERα positive breast tumor (Qin et al., 2018). Peptides 12a (HKIKHRLLQ) and 13 (Rcyclo (DILDap) RLLQ) as reported by Zhao et al. showed significant binding to ERα receptor (the value of KD were 118 nM and 85 nM respectively) (Zhao et al., 2016) (Figure 2).

The HER2 positive accounts for over 20% in all breast cancer patients (Schrama et al., 2006; Okines and Cunningham, 2010). This type of breast cancer has poor prognosis, strong invasiveness, and a high recurrence rate (Slamon et al., 1987), so drugs for targeted therapy of HER2 positive breast cancer are good for HER2 positive patients mostly in the early stages of their disease. Du et al. designed peptides DH6 (YLFFVFER) and RDH6 (REFVFFLY) conjugated with PEG that showed good metabolic stability and specially targeted HER2 positive tumors, while not HER2 negative tumors (Du et al., 2020). Stefanick et al. (Stefanick et al., 2015) reported targeting HER2 positive tumors with peptides HERP5, HRAP, KAAYSL, and AHNP for cellular uptake, in which peptide KAAYSL had the maximum tumor uptake. Kim et al. (Kim et al., 2020) prepared liposomal nanoparticles with DOX-lipid conjugates and HER2pep (selected form peptides reported by Stefanick et al. (Stefanick et al., 2015), the sequence is YCDGFYACYMDV) lipid conjugates, and the nanoparticles at 0.5% peptide density enhanced ∼2.7 fold and ∼3.4 fold the uptake of HER2 positive tumors with EG8 and EG18 linker over non-targeted nanoparticles, respectively. A9 non-apeptide (WAVQNTDAV) site-specifically conjugated with diethylene triamine penlaacetic acid (DTPA) and radiolabeled with 111In (Honarvar et al., 2018) was conducted for non-invasive imaging of HER2 positive tumors, and the results showed that DTPA-A9 had sufficient *in vivo* stability. Martyna Michalska et al. (Michalska et al., 2016) designed peptide LTVSPWY functionalized alloyed CuInZnxS2+x quantum dots as a fluorescent nanoprobe of HER2-positive breast tumors, and peptide LTVSPWY worked for targeted binding to HER2. Peptide 51(ATWLPVPVVGYFMASA) selected by Larimer and Deutscher (Larimer and Deutscher, 2014) was used to target BT-474 human breast cancer and other HER2 positive cancers in order to reduce the non-target accumulation in other tissues (Figure 2).

Epidermal growth factor receptor (EGFR), a transmembrane receptor, is an attractive target for strategies of breast cancer treatment (Gonzalez-Conchas et al., 2018). Its overexpression is correlated with reduced overall survival and disease-free and poor prognosis in breast cancer patients (Herbst and Shin, 2002). Peptide 1-D03 (MEGPSKCCYSLALSH) selected from a phage microlibrary by Larimer et al. (2014) showed high specificity for only MDA-MB-435 cells according to EGFR. Peptide GE11 (YHWYGYTPQNVI) can specifically target EGFR and bind to one region of EGFR (Genta et al., 2017; Li et al., 2021). Hailing et al. synthesized GE11 modified polylactic-co-glycolic acid/D-a-Tocopheryl polyethylene glycol 1,000 succinate nanoparticles for delivering salinomycin to breast cancer. The results showed that these nanoparticles potently enhanced the therapy efficacy to EGFR overexpressed breast cancer (Hailing et al., 2022) (Figure 2).

Different sequences of peptides have been designed in order to treat breast cancer according to specific affinity to breast cancer overexpressed receptors in order to develop an ideal peptide for targeting breast cancer now. These overexpressed receptors mainly include HSP gp96, integrins, gC1q receptor, neuropilin-1 receptors, keratin 1 receptor, galectin-3 receptor, ERs, HER2 receptor and EGFR. These targeting peptides are numerous, so we selected some representative peptides to illustrate in Figure 2 and Table 1, such as peptide p37 targeting HSP gp96, peptides RGD and RGDK-octa-arginine targeting integrins, linear peptide TT1 targeting gC1q receptor, peptides tLyP-1 and CK3 targeting neuropilin-1 receptor, linear peptides p160 and p18 targeting keratin 1 receptor, peptide G3-C12 targeting galectin-3 receptor, peptides 12a and 13 targeting ERs, peptides DH6, RDH6, KAAYSL, HER2pep, A9, LTVSPWY and 51 targeting HER2 and peptides 1-D03 as well as GE11 targeting EGFR. They all showed high binding to their corresponding receptors. There are also many challenges of targeting peptides including *in vivo* instability, low affinity to target, high non-target tissues uptakes and low internalization (Ahmadpour and Hosseinimehr, 2019).

**TABLE 1:** Summary of the major peptides against breast cancer with respective targets.

Peptide	Sequence	Target/Ref.
P37	LNVSRETLQQHKLLKVIRKKLVRKTLDMIKKIADDKY	gp96 Liang et al. (2021)
RGD/RGDK-octa-arginine	RGD/RGDK-octa-R	Integrin Garate et al. (2015); Hazeri et al. (2022)
TT1	AKRGARSTA	gC1q Simón-Gracia et al. (2018))
tLyP-1/CK3	CGNKRTR/CLKADKAKC	neuropilin-1 Zhu et al. (2018); Feng et al. (2014)
p160/p18	VPWMEPAYQRFL/WXEAAYQRFL	keratin 1 Askoxylakis et al. (2005); Shahin et al. (2013)
G3-C12	ANTPCGPYTHDCPVKR	galectin-3 Kumar and Deutscher (2008)
12a/13	HKIKHRLLQ/Rcyclo (DILDap) RLLQ	ER Zhao et al. (2016)
DH6/RDH6/KAAYSL/HER2pep/A9/LTVSPWY/51	YLFFVFER/REFVFFLY/KAAYSL/YCDGFYACYMDV/WAVQNTDAV/LTVSPWY/ATWLPVPVVGYFMASA	HER2 Du et al. (2020); Kim et al. (2020); Stefanick et al. (2015); Honarvar et al. (2018); Larimer et al. (2014)
1-D03/GE11	MEGPSKCCYSLALSH/YHWYGYTPQNVI	EGFR Larimer et al. (2014); Hailing et al. (2022)

### 2.2 CPPs for anticancer drug vectors

CPPs along with various drugs penetrating into cytomembranes by direct penetration or endocytosis (Zorko and Langel, 2005; Xu et al., 2019) enhance the anticancer efficacy of difficultly penetrable molecules for breast cancer therapy (Fukunaga et al., 2019; Tesauro et al., 2019). Nam et al. (Nam et al., 2021) developed a pH-activatable CPP dimer LH2 (monomer was LHHLCHLLHHLCHLAG) to deliver paclitaxel into triple negative MDA-MB-231 cells on account of the weak acidic condition around the tumor, which was about pH 6.0 (Fan et al., 2016) tested the CPP C (GPGLWERQAREHSERKKRRRESECKAA) conjugated with peptide SP90 having breast tumor homing ability, especially for triple negative MDA-MB-231 cells. SP90-C revealed well targeted delivery function for anticancer drugs. Park et al. (2021) synthesized anticancer drugs targeting the weak acidic condition of the breast tumor by conjugating cyclic CPP (cCPP, CWRWRKWRWR) with a targeting peptide (TP1 or TP2) and anticancer drug cabazitaxel (CBT). TP1-cCPP-CBT revealed antiproliferative activity on MDA-MB-231 and MCF-7 cells. 5(6)-Carboxyfluorescein (FAM) used as a control for confocal microscopy and cellular uptake studies was conjugated with TP1-cCPP or cCPP. The CPP gH625 (HGLASTLTRWAHYNALIRAFC) synthesized by Ben Djemaa et al. was used for CPP-capped stealth magnetic siRNA nanovectors (CS-MSN) to deliver siRNA for treating triple negative MDA-MB-231 cells. Peptide gH625 facilitated siRNA into the cell and promoted it escaping from endosomes (Ben Djemaa et al., 2019). Ben Djemaa et al. (Ben Djemaa et al., 2018) also developed CPP-capped stealth-fluorescent nanoparticles (CS-FNP) functionalized with gH625 and cationic polymers nanovectors to treat triple negative MDA-MB-231 cells; the MDA-MB-231 cells had higher uptake of optimized nanovectors than CS-FNP. CPP GALA (WEAALAEALAEALAEHLAEALAEALEALAA) designed to interact with cytomembranes at acidic pH (Subbarao et al., 1987; Li et al., 2004) and the tumor homing linear pentapeptide (CREKA) modified redox-responsive complex co-delivered siRNAs for triple negative MDA-MB-231 cell therapy. The results showed that the CREKA and GALA modified with redox-responsive complex had excellent transfection efficiency and protected siRNA from the degradation of RNA enzymes (Zhang et al., 2021). Wang et al. (2020) designed tumor homing peptide tLyP-1 (CGNKRTR) which penetrated inside cells through the CendR pathway mediated by neruopilin-1 modified micelles to co-deliver chemotherapeutic and TRPA-1 inhibitor for treatment of triple negative MDA-MB-231 cells. The results revealed that peptide tLyP-1 improved the tumor-targeting delivery of micelles. The major CPPs are shown in Table 2.

**TABLE 2:** Summary of the major cell penetrating peptides as anticancer drugs vectors for breast cancer therapy.

CPPs	Sequence/Ref.
LH2	(LHHLCHLLHHLCHLAG)_2_ Nam et al. (2021)
C	GPGLWERQAREHSERKKRRRESECKAA Fan et al. (2016)
cCPP	CWRWRKWRWR Park et al. (2021)
gH625	HGLASTLTRWAHYNALIRAFC Ben Djemaa et al. (2019)
GALA	WEAALAEALAEALAEHLAEALAEALEALAA Li et al. (2004)
tLyP-1	CGNKRTR Wang et al. (2020)

The TNBCs are difficult to treat and chemotherapy drugs commonly have low internalization for the TNBCs cells (Jamdade et al., 2015; Jain et al., 2020). According to the above researches, we found that CPPs were more studied for enhancing the cell penetration of chemical drugs or siRNA to treat TNBCs. To increase the internalization, many CPPs such as peptide dimer LH2, peptide C, cCPP, peptide gH625, peptide GALA and peptide tLyP-1 were selected for delivering chemical drugs or siRNA to treat TNBCs. However, several inherent properties limit the clinical applications of CPPs (Nam et al., 2021). One limitation is that effective penetrating concentrations of CPPs are generally above micromolar and these concentrations are difficult for CPPs *in vivo* due to instability (Milletti, 2012; Guidotti et al., 2017). Another limitation is that some CPPs indiscriminately penetrate into almost all cytomembrane due to the electrostatic and hydrophobic interactions between CPPs and cell membranes inducing the CPP internalization (Reissmann, 2014; Shi et al., 2014). Therefore (Kim et al., 2018), targeting delivery and local administration have been selected for clinical applications of CPPs (Dissanayake et al., 2017; Pescina et al., 2018). Many studies selected pH-activatable CPPs as delivery vectors for targeting the weak acidic condition around the tumor or combined CPPs with targeting peptides for specific delivery of drugs to treat tumors with low side effects.

## 3 Peptide-based cancer vaccines

### 3.1 Peptide-based breast vaccines ongoing clinical trails

Therapeutic cancer vaccines are at the forefront of current medical development (Sobhani et al., 2022). There are several types of cancer vaccines including nucleic acid-based vaccines (both DNA and RNA vaccines), protein vaccines, inactivated patient-derived tumor cells and purified tumor antigens vaccines. For breast cancer, a total of 44 therapeutic cancer vaccines are currently being investigated with ongoing clinical trials as of 23rd June 2021 (Corti et al., 2022), of which, 30 clinical trials are open to TNBC patients, 21 clinical trials are enrolling HER2-positive breast cancer patients while there are only 15 trials with hormone receptor-positive breast cancer as inclusion criteria. Among these breast cancer vaccine ongoing clinical trials, peptide-based vaccines are in the majority (20, 45.5%). de Paula Peres et al. (de Paula Peres et al., 2015) reviewed 13 types of main peptide-based vaccines in breast cancer studied on phase III, phase II, phase I/II and phase I clinical trials, including peptides E75 (KIFGSLAFL), GP2 (IISAVVGIL), AE37 (LRMKGVGSPYVSRLLGICL), P15 (FLAEDALNTV), P14 (NLMEQPIKV), P13 (YLIELIDRV), P7 (ILDDIGHGV), P5 (ALMEQQHYV), P4 (FLYDDNQRV), P3 (KLDVGNAEV), MUC1-KLH conjugate plus QS-21 (KLH-MBS-CVTSAPDTRPAPGSTAPPA HGVTSAPDTRPA), MFP (PDTRPAPGSTAPPAHGVTSA) and L-BLP25 (STAPPAHGVTSAPDT RPAPGSTAPP) (Table 3). In addition, peptide vaccines including those of different delivery vectors and adjuvants have been implemented (Antonarelli et al., 2021) for the main reason of poor immunogenicity of single-peptide vaccines.

**TABLE 3:** Summary of the vaccination based on peptide clinical trials on breast cancer patients. (de Paula Peres et al., 2015).

Vaccine	Peptide	Gene	Sequence	Target(protein)
NeuVax^**TM**^	E75(nelipepimut-S)	HER2	KIFGSLAFL	Her-2/neu
	GP2	HER2	IISAVVGIL	Her-2/neu
	AE37	HER2	LRMKGVGSPYVSRLLGICL	Her-2/neu
Depovax^**TM**^ (DPX-0907)	P3	BAP31	KLDVGNAEV	BAP31/CDMproteinTopoisomeraseII a
	P4	TOP2A	FLYDDNQRV	Integrin β8subunitprecursorAbl-bindingprotein 3CTACE/ADAM-17
	P5	ITGB8	ALMEQQHYV	JunctionplakoglobinEDDRI
	P7	ABI_2	ILDDIGHGV	
	P13	ADA17	YLIELIDRV	
	P14	JUP	NLMEQPIKV	
	P15	DDR1	FLAEDALNTV	
	MUC1-KLH conjugate plus QS-21	MUC1	KLH-MBS-CVTSAPDTRPAPGSTAPPAHGVTSAPDTRPA	Mucin-1
	Oxidized mannan-MUC1 (MFP)	MUC1	PDTRPAPGSTAPPAHGVTSA	Mucin-1
Stimuvax	L-BLP25	MUC1	STAPPAHGVTSAPDTRPAPGSTAPP	Mucin-1

### 3.2 Peptide vaccines combined with vectors or adjuvants

**FIGURE 3:**
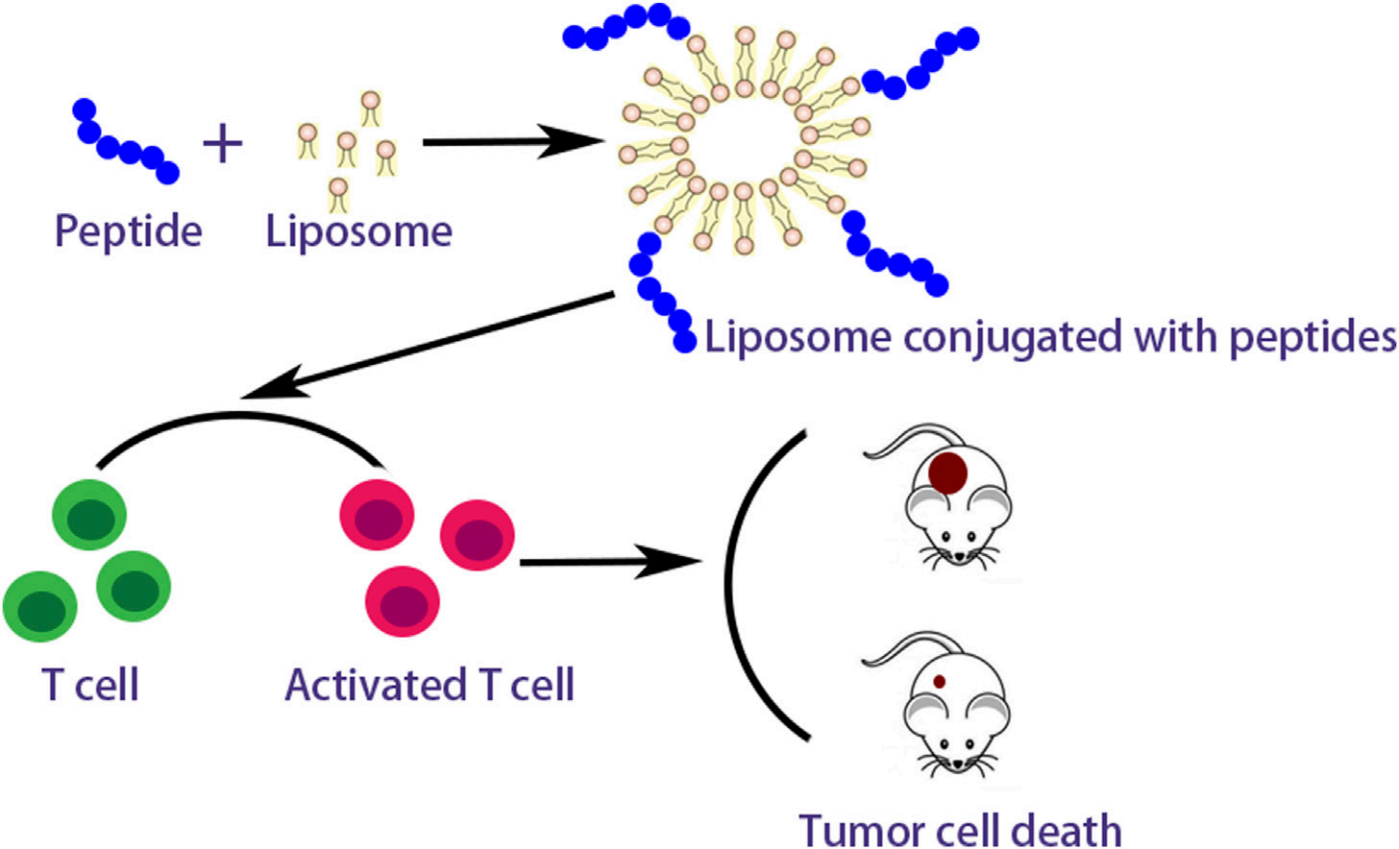
Action mechanism of peptide vaccines combined with vectors.

Many researches on peptide-based breast cancer vaccines in combination with vectors or adjuvants are currently proceeding. Zamani et al. (2020) comninated long multi-epitope peptide E75-AE36 (Ac-CGGGKIFGSLAFLAAAGVGSPYVSRLLGICL) with peptide PADRE (AKFVAAWTLKAAA) to form nanoliposomal vaccine and estimated its immunogenicity for the breast cancer of mice. The results revealed this nanoliposomal vaccine obviously enhanced the production of IFN-γ and responses of CD4^+^ and CD8^+^ T cells. Zamani et al. (2022) also studied another nanoliposomal-based peptide vaccine composed of peptide AE36 (Ac-CGGGVGSPYVSRLLGICL) and peptide PADRE conjugated with monophosphoryl lipid A (MPL) adjuvant. The results showed that an effective immune response was triggered by this peptide vaccine in breast cancer mice with the TUBO model and stimulated more robust Th1 immune responses. Peptide AE36 was also coupled with liposomes containing DOPE, 1,2-dioleoyl-3-trimethylammonium propane (DOTAP) and cholesterol, by Barati et al. (Barati et al., 2022). Such formulations could effectively induce the responses of CD8^+^ and CD4^+^ T cells. Zamani et al. also investigated the combination therapies with peptide E75 (Ac-CGGGKIFGSLAFL) and DOX which could synergistically potentiate IFN-γ, CD8^+^, and CD4^+^ T cell responses (Zamani et al., 2020a). Peptide E75 also coupled with polyactin A (PAA) usually selected to treat impaired immunity in China could significantly increase positive rates of CD8^+^ and CD4^+^ T lymphocyte and potentiate IFN-γ production compared with pure peptide E75 (Wang et al., 2018). A nano-liposomal vaccine containing peptide P5, MPL, and peptide PADRE adjuvant showed prominent anticancer effects in HER2 positive breast cancer and effectively activated CD8^+^ T cell immune response (Zamani et al., 2019). Peptide P5 loaded in cationic nanoliposome composed of polyriboinosinic polyribocytidylic acid [Poly (I:C)] and DOTAP-cholesterol induced strong antitumor responses both *in vivo* and *in vitro* (Talesh et al., 2016). The peptide vaccines combined with vectors or adjuvants reveal the action mechanism as showed in Figure 3.

### 3.3 Novel peptide vaccines

Many novel peptides were studied as breast cancer vaccines except combining with vectors and adjuvants as shown in Table 4. Mahdavi et al. reported peptide 249 (GDLTNRCTMEEKPMEK) as a potential peptide-based vaccine derived from insulin-like growth factor-1 (IGF-1) receptor in breast cancer (Mahdavi et al., 2017) and peptide 249 could excellently bind to Major Histocompatibility Complex (MHC) class Ⅱ and Ⅰ and T-cell. They also synthesized peptide 1412 (QPEQQETKKEEQ) and this peptide showed outstanding binding activity to most of the applied MHCs (Mahdavi and Moreau, 2016). Peptide E39 (EIWTHSYKV) (Vreeland et al., 2018) was combined with peptide E39’ (EIWTFSTKV), an attenuated version of E39. Immune analyses suggested that the optimal cytotoxic T lymphocyte population was induced by administering with E39 and E39’ in t urn. Peptide ErbB-2266-296 (CGPSLLHCPALVTYNTDTFESMHNPEGRYTFGASCV) binding to the native ErbB-2 protein by mimicking the pertuzumab for activating B cells and ovalbumin peptide OVA323-339 (ISQAVHAAHAEINEAGR) as a T cell epitope were connected as a liposomal system for inducing anti-ErbB-2 antibody response (Wallis et al., 2020). The mice tests showed that humoral immune response activated by peptides ErbB-2266-296 and OVA323-339 raised 7.3-fold and they also induced 96% tumor cell death *in vitro*. Servín-Blanco et al. developed a Variable Epitope Library (VEL) and identified long peptides 45A2L (AGDAEYXRAYLXGECVEXLRXYLXLGNXTLLRXDXPKAHVTYHPRS) and QA2L (GPHXLRYFXTAVXWPGLVXPRFIXVGYVXDTQFV) as well as their wild type peptides PPE15L (MDFGXLPPEXNSXRMYAGXGAXPMMAAXAAWNGLAXELGTXAASY) and PGRS (LFXNGGAGGQGGXGGXGGXGGXGGXGMAXGPAGGTGGIGXIGGIG) (the “X” represents any of 20 amino acids) (Servín-Blanco et al., 2018). They observed that 45A2L, QA2L, PPE15L, and PGRS significantly activated T cell for inhibiting tumors and reduced metastatic lesions in lungs of immunized mice. Ganneau et al. synthesized glycopeptide dendrimer MAG-Tn3 (containing peptide TT, its sequence is QYIKANSKFIGITEL) for an anticancer vaccine candidate for human use (Ganneau et al., 2017). They found that MAG-Tn3 induced Tn-specific antibodies that killed Tn-positive cancer cells of human and MAG-Tn3 was now being evaluated for cancer vaccine in breast cancer patients (phase I clinical trial).

**TABLE 4:** Summary of the major peptide-based vaccines at early stage research for breast cancer therapy.

Peptides	Sequence/Ref.
249	GDLTNRCTMEEKPMEK Mahdavi et al. (2017)
1,412	QPEQQETKKEEQ Mahdavi and Moreau (2016)
E39/E39′	EIWTHSYKV/EIWTFSTKV Vreeland et al. (2018)
ErbB-2266-296/OVA323-339	CGPSLLHCPALVTYNTDTFESMHNPEGRYTFGASCV/ISQAVHAAHAEINEAGR Wallis et al. (2020)
45A2L/QA2L/PPE15L/PGRS	AGDAEYXRAYLXGECVEXLRXYLXLGNXTLLRXDXPKAHVTYHPRS/GPHXLRYFXTAVXWPGLVXPRFIXVGYVXDTQFV/MDFGXLPPEXNSXRMYAGXGAXPMMAAXAAWNGLAXELGTXAASY/LFXNGGAGGQGGXGGXGGXGGXGGXGMAXGPAGGTGGIGXIGGIG (the “X” represents any of 20 amino acids) Servín-Blanco et al. (2018)
MAG-Tn3	QYIKANSKFIGITEL Ganneau et al. (2017)

Cancer vaccines should be tumor specific and well tolerated and they have well-known advantages over chemotherapy, so peptide-based cancer vaccines are promising for prevention and treatment breast cancer. The studies of main peptide-based vaccines such as E75, GP2, AE37, P3, P4, P5, P7, P13, P14, P15, MUC1-KLH conjugate plus QS-21, MFP, and L-BLP25 have been used on phase III, phase II, phase I/II and phase I clinical trials. In the last 2 decades, the researches of breast cancer vaccines showed poor clinical trial results and the immunogenicity of single-peptide vaccines was poor. However, breast cancer vaccines have been widely considered as candidates for the technological advancements by the COVID-19 vaccination campaign (Corti et al., 2021; Dafni et al., 2021). For inducing long-lasting immune responses, cancer vaccines could be combined with other treatment modalities (Dafni et al., 2021). Peptides connected with different delivery vectors and adjuvants as breast cancer vaccines have been widely studied. The delivery vectors are evolving towards non-degradable and polymeric nanoparticles, virus-like particles and lipid nanoparticles (Antonarelli et al., 2021), with liposomes showing superior performance in increasing cellular uptake to enhance vaccine immunogenicity and helping to deliver peptide-based vaccines to the lymph nodes (Watson et al., 2012). Peptide-based vaccine monotherapy has mainly focused on premalignant cancer or the (neo) adjuvant setting while peptide-based vaccines combined with anticancer drugs have been used in late-stage tumors. Peptide-based breast cancer vaccines have shown good prospects and further understanding of cancer vaccines as well as technological advancements in their preparation and use are important.

## 4 Peptides for anti-cancer drugs

### 4.1 Peptides disrupting the cellular membrane

Melittin (GIGAVLKVLTTGLPALISWIKRKRQQ) constituting about 50% of dry weight of honey bee venom (Terwilliger and Eisenberg, 1982) showed good anticancer activity in breast cancer models by disrupting the cellular membrane. For the molecular mechanisms of melittin as anticancer agent, Duffy et al. demonstrated that melittin induced cancer cell death, especially in TNBC and HER2 breast cancers, by inducing that the EGFR and HER2 could not be phosphorylated in the cell membrane of breast cancer cells (Duffy et al., 2020). While melittin delivered in vectors is preferred due to its rapid lytic effects (Sorolla et al., 2020), Daniluk et al. designed melittin carried by nanographene oxide or nanodiamonds and found that nanographene oxide vector could increase the anticancer activity of melittin for breast cancer and nanodiamonds are able to protect normal cells from the destroy of melittin (Daniluk et al., 2019). Melittin complexed with micelles modified with estrone held steady against trypsin digestion and the micelles conjugated with estrone showed more than 6-fold increase in uptake of ER positive MCF-7 cells relative to the TNBC cell line MDA-MB-231 as well as high anticancer efficacy (Raveendran et al., 2020; Motiei et al., 2021). For targeted co-delivery of melittin with miR-34a, Motiei et al. synthesized a nanocarrier composed of polyethyleneimine shell modified with folic acid, dextran sulfate as a complexing agent and polyglutamate grafted chitosan core (Motiei et al., 2021). The results revealed that the deaths of cancer cells were obviously increased by 54% by co-delivery of melittin and miR-34a. Melittin was used for treating breast cancer brain metastases (BCBM), by conjugating AMD3100, a small molecule antagonist of CXCR4 highly expressed in BCBM, with poly(lactone-co-β-amino ester) nanoparticles and melittin delivered *via* AMD3100 nanoparticles effectively inhibited tumor growth in mice of BCBM model (Zhou et al., 2020). In addition, melittin has good immunoregulatory activity such as augmenting Th1 cell function (Qiubo et al., 2000). Liu et al. prepared mutant interleukin-2 (MIL-2), one of the most successful cytokines for tumor immunotherapy and melittin to form a bifunctional fusion protein melittin-MIL-2 (Liu et al., 2016). The results showed that the melittin-MIL-2 could induce obvious antitumor and immune stimulation effects.

### 4.2 Peptides suppressing the activity of transcription factors

Interference peptides (iPeps), mutational transcription factor versions, are inert and able to inhibit the transcriptional program by sequestering their binding partners tight (Sorolla et al., 2020). Beltran et al. (2014) designed seven iPeps including iPep697Δ (KKKRKVAPAAVYCTRYSDR), iPep697 (KKKRKVWPAWVYCTRYSDR), iPep682 (KKKRKVPLVWPAWVYCTRYSDRPS), iPep624 (KKKRKVTDSQQPLVWPAWVYCTRYSDRPS), iPep624W1ΔA (KKKRKVTDSQQPLVAPAWVYCTRYSDRPS), iPep624W2ΔA (KKKRKVTDSQQPLVWPAAVYCTRYSDRPS) and iPep624ΔHEX (KKKRKVTDSQQPLVGAAGAGCTRYSDRPS). These iPeps could target engrailed 1 (EN1) on account of their sequences which comprises the flanking protein sequences from the N terminus of the homeodomain (HD) and specific hexamotif of comprising EN1. The results showed that iPep624 and iPep682 rapidly mediated a strong apoptotic response in breast cancer cell line SUM149PT which overexpressed EN1. iPep697 and iPep697Δ were internalized by cell in ˂2 min and reached a high land 40 min later (Beltran et al., 2014). Sorolla et al. synthesized active EN1-iPep (KKKRVPLVWPAWVYCTRYSDR) as well as mutant EN1-iPep (KKKRVPLVAPAAVYCTRYSDR) and engineered two iPeps with four peptide RGDs including RGD1 (HGRGDLGRLKK), RGD2 (YTSGDQKTIKS), RGD3 (NLRGDLQVLAQ) as well as RGD4 (RGRRGDLATIHG). EN1-RGD-iPeps was applied to functionalize the docetaxel nanoparticles for promoting tumor specific targeting. The results revealed that EN1-RGD-iPeps reduced cell viability and induced apoptosis in TNBC cells without showing toxicity and EN1-RGD-iPeps-mediated docetaxel nanoparticle increased tumor accumulation *via* integrins and intravenous injection (Sorolla et al., 2019). They also designed active EN1-iPep (KKKRKVPLVWPAWVYCTRYSDR) and mutant EN1-iPep (KKKRKVPLVAPAAVYCTRYSDR) (Sorolla et al., 2016). The *in vitro* cell toxicity assays showed high selective toxicity of EN1-iPep in human basal-like SUM149PT cells while not in MCF10A cells, a normal breast epithelial cell line. Furthermore, EN1-iPep could improve the inducing apoptosis function of docetaxel by synergistic pharmacological action when they were co-delivered by poly(acrylic acid) modified poly(glycidyl methacrylate) nanoparticles.

### 4.3 Peptides for treating resistant breast cancer

The resistance of anticancer drugs is a critical problem limiting the extended use of chemotherapy drugs in breast cancer therapy (Cabeza et al., 2015). In almost every patient whose cancer shows resistance, it is inevitable that the relapse and metastasis of tumors appear after endocrine treatment (Mazumder et al., 2021). The two resistance mechanisms include the intrinsic resistance of tumors before administration of chemotherapy and the generated resistance during therapy of tumors previously suppressed by chemotherapy drugs (Wilson et al., 2006). The latter resistance is even more problematic due to its difficult predictability and multidrug resistance (MDR), many different chemotherapy drugs resisted simultaneously. There are many factors influencing drug resistance, including overexpression of ABC transporters, drug inactivation, changes in drug targets, apoptotic dysregulation, cancer stem cells and epithelial-to-mesenchymal transitions (Faruk, 2021).

The efflux mechanism in resistant breast cancer is usually achieved by overexpression of ABC transporters such as P-glycoprotein (P-gp) which can lower drug accumulation inside the cell by removing the anticancer drugs out of cells is the most popular resistance mechanism (Seelig and Gatik-Landwojtowicz, 2005). Correspondingly, many strategies are aimed at avoiding the P-gp efflux in order to overcome resistance. Mozaffari et al. synthesized conjugates [R5K]W7A-DOX and [R5K]W7C-S-S-DOX with a glutarate linker and a disulfide linker, respectively. They both contained DOX and [R5K]W7A, a peptide including linear W7A and cyclic R5K (Mozaffari et al., 2021). The anticancer results showed that [R5K]W7A-DOX conjugate had 16-fold higher activity against DOX-resistant breast cell line MDA-MB-231R compared to free DOX. The MTT results revealed that [R5K]W7A-DOX conjugate (5 µM) had no significant cytotoxicity to heart muscle cells and kidney cells compared to free DOX (5 µM) obviously increasing the cell apoptosis and destroying the morphology of the cells after 72 h of incubation. Prolonging intracellular retention is also important towards alleviating cancer cell MDR by suppressing the P-gp efflux. Nanocarriers have been showed to be an efficient approach to increase retention of anticancer drugs, both inside the tumor cells and in circulation (Yuan et al., 2015). Zhang et al. proposed a strategy based on the peptide Nap-GFFpYK covalently bound to etoposide in order to increase the intracellular retention and the water solubility (Zhang et al., 2020). Compared to free etoposide, Nap-GFFpYK-etoposide conjugate showed a 20 -fold inhibitory activity on these cells with artificially overexpressed MDR1. The phosphorylation of P-gp significantly enhanced the MDR phenotype of the breast cells, and protein kinase C (PKC), an isozyme family, could phosphorylate the linker region of P-gp (Orr et al., 1993). PKC-pseudosubstrate peptides 1–7 were synthesized and their sequences were P1 (NmFARKGALRQ), P2 (FARKGALRQ), P3 (NmRFARKGALRQKNV), P4 (RFARKGALRQKNV), P5 (NmRKRTLRRL), P6 (RKRTLRRL), and P7 (NmNDSRSSLIRKR), respectively. In these PKC-pseudosubstrate peptides, P1 revealed the most likely reversing MDR mechanism of inhibition of P-gp phosphorylation (Gupta et al., 1996). Zhi et al. designed a novel compound, LAX, composed of tetrapeptide (GFLG), DOX and lipoid acid (Zhi et al., 2019), in which tetrapeptide was a trigger of lysosomal protease cathepsin B existing inside most tumor cells (Li et al., 2014). They selected gold nanorods (AuNRs) as a vector to deliver LAX to form an enzyme-responsive nanomedicine AuNRs-LAX. In addition, AuNRs-LAX could bypass the P-gp efflux due to their size, which was much larger than the active binding site of P-gp (Browning et al., 2018). The results revealed that the AuNRs-LAX treatment reduced the drug resistance index from greatly high 955.0 to 1.7 in MCF-7/ADR cell resisting DOX (Zhi et al., 2019).

In addition to efflux mechanism, apoptotic dysregulation is also an important factor of drug resistance. One important contributor of apoptotic dysregulation was mitogen-activated protein kinase (MAPK) signaling cascade. Extracellular signal regulated kinases (ERK), a principal protein molecule in MAPK cascade, stimulates cell survival and growth by initiating the transcription of specific genes (Choi et al., 2008). Within this context, Sheng et al. prepared peptide T10 (HAIYPRH) connected with peptide MPKKKPTPIQLNP, a ERK peptide inhibitor, by a thiol spacer (GGCG) to form peptide T10-ERK(HAIYPRHGGCGMPKKKPTPIQLNP) and conjugated DOX with peptide T10-ERK for the reversal study in drug resistant breast cancer (Sheng et al., 2016). The results revealed that the additive effect of ERK peptide inhibitor together with DOX-T10 led to less susceptibility and greater efficacy to drug resistance. To reverse apoptotic dysregulation, initiating the lysosomal apoptosis pathway was also efficient. Tetrapeptide GFLG, sensitive to cathepsin B, was also used to link mPEGylated dendron with DOX to self-assemble conjugate DOX-GFLG-mPEGylated dendron and this conjugate could enhance cell death mediated by lysosomal according to inducing cathepsin B in the cytoplasm (Wang et al., 2020). Proliferating cell nuclear antigen (PCNA) plays a central function including protein degradation, cell cycle regulation, chromatin remodeling, DNA methylation, translesion DNA synthesis, DNA repair and DNA replication (Strzalka and Ziemienowicz, 2011). Cancer-associated PCNA (caPCNA) has an 8 amino acid peptide caPeptide (CLGIPEQEY) on a binding domain. Lingeman et al. improved the endogenous expression of caPeptide by a tetracycline responsive promoter in MDA-MB-231 cells (Lingeman et al., 2014). The results showed that caPeptide combined with cisplatin generated a noticeable increase in the apoptosis of MDA-MB-231 cell line resisting cisplatin. Ras-related nuclear protein (Ran) controlled the cellular responses to DNA damage and cell cycle rate by regulating nuclear import and export pathways. When the Ran concentration reached a threshold, DNA damage in tumor cells was successfully repaired. Haggag et al. have reported a specific Ran guanine nucleotide exchange factor inhibitory peptide (RAN-IP, its sequence is CAQPEGQVQFK) which could block the interaction between Ran and Ran guanine nucleotide exchange factor in MDA-MB-231 breast cancer cells to inhibit RanGTP formation (Haggag et al., 2019). Ran-IP and DOX were co-delivered by a liposomal delivery system using a thin-film rehydration technique. Ran showed good reversing of the Ran-expression-mediated MDR activity and obviously enhanced the sensibility of tumor-bearing mice to DOX (Haggag et al., 2020).

One efficient strategy of overcoming resistance is bypassing resistance mechanisms by specific binding to new targets. Accordingly, tetra-branched peptides could bind to membrane heparan sulfate proteoglycans or the low density lipoprotein receptor family at sulfated glycosaminoglycan chains or different endocytic receptors respectively though their branched structure which contained human neurotensin (NT4, the sequence is (ELYENKPRRPYIL)_4_) (Brunetti et al., 2016), were used to conjugate with methotrexate (MTX) for MTX-resistant human breast cancer therapy. The results showed that MTX-conjugated NT4 bypassed drug resistance and was efficiently internalized by MTX-resistant human breast cancer cells (Depau et al., 2017). Neuropeptide Y (NPY) analogue [F7,P34]-NPY (YPSKPDFPGEDAPAEDLARYYSALRHYINLITRPRY) was toxic on breast cancer by the human Y1-receptor (hY1R) mediated pathway. Böhme et al. introduced two MTX at positions 4 and 22 of [F7,P34]-NPY and this conjugate was more effective than MTX on MTX-resistant cells (Böhme et al., 2016). Endocrine resistance was a common obstacle in therapy for ERa-positive breast cancer and prohibitin 2 (PHB2) could interact with ERa to inhibit ERa transcriptional activity. Guanine nucleotide-exchange protein 3 (BIG3) inhibited by brefeldin A could activate the ERa signaling pathways by capturing PHB2 in most breast cancer (Kim et al., 2009). Yoshimaru et al. synthesized a negative peptide ERAP (11R-GGG-QMLSDLTLQLRQR) which could release PHB2 from BIG3 and peptide ERAP treatment enhanced tamoxifen responsiveness in ERa-positive breast cancer cells (Yoshimaru et al., 2013). Annexin 1 (Anxa 1) was an apparently expressed biomarker on the surfaces of tumor vasculature ECs (Hatakeyama et al., 2011). In mice of null Anxa 1, the lack of angiogenesis significantly suppressed tumor growth (Chen et al., 2013). A peptide designated IF7 (IFLLWQR) could bind to Anxa 1 with high affinity and specificity so Yu et al. synthesized a nano-DDS of IF7-PTX-NP by conjugating peptide IF7 with paclitaxel. The results showed that IF7-PTX-NP could significantly induce necrosis of the tumor tissues and apoptosis of the tumor vascular endothelial cells with no obvious toxicity to the mice (Yu et al., 2015).

### 4.4 Other anticancer peptides

**FIGURE 4:**
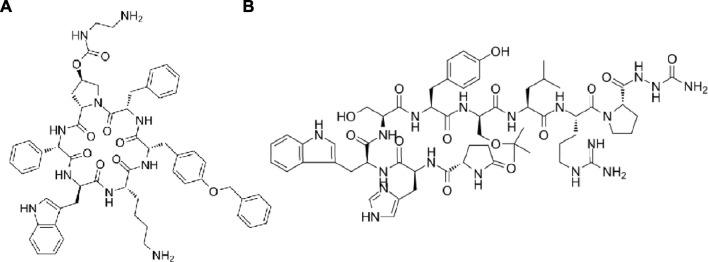
**(A)** The structure of pasireotide. **(B)** The structure of goserelin.

Long non-coding RNAs (lncRNAs) which were longer than 200 nucleotides were non-protein coding transcripts and regulated cell proliferation as well as invasion (Li et al., 2017). An lncRNA LINC00908 which specially translated in TNBC cell line MDA-MB-231 encoded a 60-aa peptide ASRPS (MTTKMRRLRPSAPSGLGQEQEAEVVEGCFPAVTETPFAPAYIKKRGGRIWSSDPRSDGEH). Peptide ASRPS reduced the expression of vascular endothelial growth factor (VEGF) by down-regulating STAT3 phosphorylation so the ASRPS expression reduced angiogenesis of TNBC (Wang et al., 2020). Peptide AFPeq (cyclo(EKTOVNOGN), O is hydroxyproline), a head-to-tail cyclized peptide developed by Jacobson et al. mimics the anti-breast cancer site of α-fetoprotein (Jacobson et al., 2014). AFPeq initially inhibited (days 0–7) and then stopped MCF-7 breast cancer xenografts growth (days 7–14) while it had no effect on liver and hepatocellular carcinoma cell line growth. In addition, AFPeq had good tolerance in mice, rats, dogs, and monkeys (Mansouri et al., 2018). There are two peptides specifically used to treat breast cancer now and they are pasireotide and goserelin. Pasireotide (Figure 4A, cyclo(Hyp(Unk)-Phg-WKY(Bn)F)) can specifically binding to somatostatin receptor subtypes sst5 and sst1,2,3 and suppress the adrenocorticotropic hormone, IGF-1 and growth hormone secretion potently (Schmid, 2008). Goserelin (Figure 4B, XHWSYSLRP) showed different mechanism of action. It can bind to gonadotrophin-releasing hormone (GnRH) receptor on anterior pituitary cells as an GnRH analogue and stimulate the anterior pituitary secreting luteinizing hormone and follicle-stimulating hormone with low proteolytic susceptibility (Parker and Schimmer, 2001).

**FIGURE 5:**
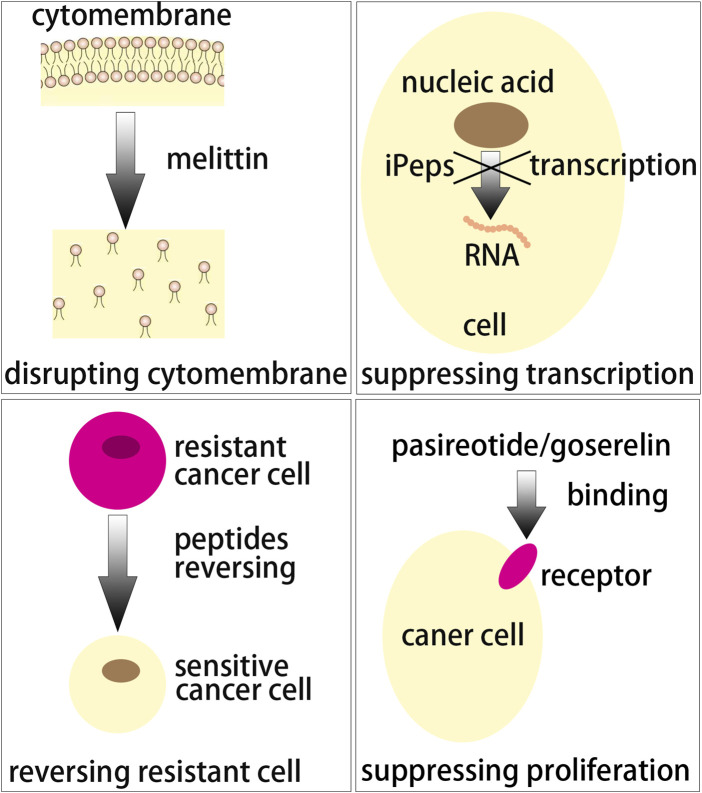
Several action mechanisms of peptide-based anticancer peptides.

Peptide drugs used to treat tumors have been limited by some factors such as serial design, structural modification and pharmic screening. There are 118 types of peptide drugs successfully applied to global clinic as of May 2022 and these peptide drugs include 7 types of diagnostic reagents. However, to put this in perspective, the number of peptide drugs is only 2% of that of all new drugs. The research of peptide drugs is currently at the forefront of medical development now due to the constant improvement of genetic technology, information technology and genomics technology. Some novel peptides showed in Table 5 were designed and synthesized to inhibit tumor growth by different mechanisms (Figure 5). Some researchers selected nanocarriers to deliver melittin and other anticancer drugs such as peptides, chemotherapeutic drugs or siRNA for breast cancer therapy. The results showed the obvious anticancer activity of melittin. Another anticancer mechanism of peptides is suppressing the activity of transcription factors. Mutational transcription factor versions iPeps could inhibit the transcriptional program by tight sequestering their binding partners. In some studies, different sequences of iPeps were developed for anticancer activity detection and many peptide sequences showed good inhibition of cancer cell growth activity. The MDR of tumors seriously affected the extended use of chemotherapy drugs in breast cancer therapy due to its difficult prediction and simultaneous resistance to many different chemotherapy drugs. The American Cancer Society estimated that over 90% cancer deaths were related to MDR in different degrees. There are many mechanisms of MDR including changes in overexpression of ABC transporters, drug inactivation, drug targets, apoptotic dysregulation, cancer stem cells and epithelial-to-mesenchymal transitions. Among these mechanisms, P-gp efflux and apoptotic dysregulation are concerned with the peptides treating MDR. Some peptides rich in arginine and tryptophan were designed to reverse or bypass P-gp efflux. These peptides were conjugated with anticancer drugs for treating corresponding resistance in breast tumors. The corresponding results showed that drugs conjugated with peptides had noticeably higher efficiency compared to free drugs. In addition, some peptides initiated a lysosomal apoptosis pathway or inhibited cell growth and survival peptide ERK were designed and synthesized. These peptides could notably improve the resistance to breast tumor deaths and reverse the MDR. In addition, other peptides were designed to treat MDR by the method of specific binding to new targets and then bypassing resistance mechanisms. There also some new peptides used for breast cancer therapy through other mechanisms such as reducing the expression of VEGF or by mimicking the anti-breast cancer site of α-fetoprotein.

**TABLE 5:** Summary of the major peptides used for breast cancer therapy.

Peptides	Sequence/Ref.
melittin	GIGAVLKVLTTGLPALISWIKRKRQQ Terwilliger and Eisenberg (1982)
iPep697Δ/iPep697/iPep682/iPep624/iPep624W1ΔA/iPep624W2ΔA/iPep624ΔHEX	KKKRKVAPAAVYCTRYSDR/KKKRKVWPAWVYCTRYSDR/KKKRKVPLVWPAWVYCTRYSDRPS/KKKRKVTDSQQPLVWPAWVYCTRYSDRPS/KKKRKVTDSQQPLVAPAWVYCTRYSDRPS/KKKRKVTDSQQPLVWPAAVYCTRYSDRPS/KKKRKVTDSQQPLVGAAGAGCTRYSDRPS Beltran et al. (2014)
EN1-iPep/Mutant EN1-iPep	KKKRVPLVWPAWVYCTRYSDR/KKKRKVPLVWPAWVYCTRYSDR/KKKRVPLVAPAAVYCTRYSDR/KKKRKVPLVAPAAVYCTRYSDR Sorolla et al. (2019)
[R5K]W7A	[R5K]W7A Mozaffari et al. (2021)
Nap-GFFpYK	Nap-GFFpYK Zhang et al. (2020)
P1/P2/P3/P4/P5/P6/P7	NmFARKGALRQ/FARKGALRQ/NmRFARKGALRQKNV/RFARKGALRQKNV/NmRKRTLRRL/RKRTLRRL/NmNDSRSSLIRKR Gupta et al. (1996)
tetrapeptide	GFLG (Zhi et al., 2019)
T10-ERK	HAIYPRHGGCGMPKKKPTPIQLNP Sheng et al. (2016)
GFLG	GFLG Wang et al. (2020)
caPeptide	CLGIPEQEY Lingeman et al. (2014)
RAN-IP	CAQPEGQVQFK Haggag et al. (2019)
NT4	(ELYENKPRRPYIL)_4_ Brunetti et al. (2016)
[F7,P34]-NPY	YPSKPDFPGEDAPAEDLARYYSALRHYINLITRPRY Böhme et al. (2016)
ERAP	11R-GGG-QMLSDLTLQLRQR Yoshimaru et al. (2013)
IF7	IFLLWQR Yu et al. (2015)
ASRPS	MTTKMRRLRPSAPSGLGQEQEAEVVEGCFPAVTETPFAPAYIKKRGGRIWSSDPRSDGEH Wang et al. (2020)
AFPeq	cyclo(EKTOVNOGN) Jacobson et al. (2014)
Pasireotide	cyclo(Hyp(Unk)-Phg-WKY(Bn)F) Herbert (2008)
goserelin	XHWSYSLRP Parker and Schimmer (2001)

## 5 Summary and future perspectives

Peptides participate in and regulate the functional activities of various systems, organs, tissues and cells in organisms. They play important roles in vital activities. Research on peptide-based anticancer drugs has attracted wide interest due to its functionalities of scalable production, good safety and adaptive nature. In clinical medication of breast cancer, peptides have been applied as promising targeting vectors, anticancer vaccines and anticancer drugs. Previous studies showed that peptides could specifically bind to different targets of breast tumor cells. In this review, we summarized nine targets of breast tumor cells including HSP gp96, integrins, gC1q receptor, neuropilin-1 receptor, keratin 1 receptor, galectin-3 receptor, ERs, HER2, and EGFR. Different peptides that were reported as delivering anticancer drugs to corresponding targets were itemized. In addition, CPPs which could penetrate cytomembranes by electrostatic and hydrophobic interactions were also used to deliver anticancer drugs into breast cancer cells. While CPPs are not able to distinguish tumor cells from normal cells, so that it is necessary to conjugate CPPs conjugated with a targeted portion. Peptide-based vaccines are one of medicines at the forefront of therapeutic cancer vaccine development. Some peptide-based vaccines for breast cancer have now been studied on phase III, phase II, phase I/II, and phase I clinical trials, including peptides E75, GP2, AE37, P3, P4, P5, P7, P13, P14, P15, MUC1-KLH conjugate plus QS-21, MFP, and L-BLP25. Some peptide-based vaccines in combination with vectors or adjuvants were designed due to the poor immunogenicity of single-peptide vaccines. Many cytotoxic peptides were also able to induce apoptosis of tumor cells with different mechanisms; for example, melittin disrupted cytomembrane and iPeps suppressed transcription to inhibit tumor growth. More importantly, peptides were reported that could suppress MDR breast cancer by reversing efflux, apoptotic dysregulation or bypassing resistance mechanisms because MDR breast cancer has obviously increased the mortality of breast cancer patients.

After 2000, a variety of peptides were discovered or designed for treating breast cancer. However, new peptides are few in clinical treatment of breast cancer. Only two anticancer peptides, pasireotide and goserelin, were approved to treat breast cancer as of May 2022. The clinical application of peptides is limited by several factors including target selection, design of sequences, structural modification, high proteolytic susceptibility and preclinical study. Firstly, anticancer peptides have exhibited weak targeted treatment for breast cancer. This is mainly because the targets of cancer cells are influenced by some genes and various signal pathways, so that anticancer peptides could not safely and effectively treat breast cancer by binding to a single target of cancer cells. Therefore, multifunctional peptides with synergistic mechanisms or targeting multiple points that will become more efficient should be developed and studied. Secondly, the design of sequences has limited the development of new peptide-based drugs. The sequences of peptides needed to be ameliorated continuously to obtain peptides suitable for efficient breast cancer therapy. For example, unstable amino acid residues needed to be replaced to avoid the isomerization, glycosylation and oxidation of peptides *in vivo*. Meanwhile, the physicochemical properties of peptides such as distribution, isoionic point and pH are required to be optimized. It is insufficient to simply design the sequences of peptide-based drugs only by computer-aided drug design technology without consideration of factors involving their use. Therefore, the sequences of new anticancer peptides have commonly originated from those of natural peptides with anticancer activities; for example, from snake venom or bee venom and bioactive peptides in the body. Thirdly, the structural modification of peptides is often necessary due to the high proteolytic susceptibility, high non-target tissue uptakes and low internalization of peptides *in vivo*. Many strategies have been carried out to overcome these intrinsic disadvantages. The catenulated structure of peptides generally shows high elasticity; accordingly, stable cyclic peptides with one loop or two loops were designed. Peptides have also been conjugated with drugs, antibodies or oligonucleotides for targeted therapy with low side effects. In addition, oral peptides modified with PEG, glycosyl, liposomes or adjuvants were also prepared to avoid proteolytic degradation *in vivo* for better bioavailability. Finally, the preclinical studies such as pharmacological, toxicological and metabolic studies which are the prerequisite of clinical trials must be considered. These preclinical studies require considerable time and effort to ensure the safety and effectiveness of anticancer peptide-based drugs.

The successful preparation of new peptide-based drugs ultimately requires investment in numerous researches, time and money, but in return, the payoffs of new peptide-based drugs are expected to be enormous. Peptide-based drugs or vaccines for breast cancer therapy hold considerable promise for future use and could become a major player in the future pharmaceutical market.

## References

[B1] Agemy L. Kotamraju V. R. Friedmann-Morvinski D. Sharma S. Sugahara K. N. Ruoslahti E. (2013). Proapoptotic peptide-mediated cancer therapy targeted to cell surface p32. Mol. Ther. 21 (12), 2195–2204. 10.1038/mt.2013.191 23959073 PMC3863797

[B2] Ahmadpour S. Hosseinimehr S. J. (2019). Recent developments in peptide-based SPECT radiopharmaceuticals for breast tumor targeting. Life Sci. 239, 116870. 10.1016/j.lfs.2019.116870 31525426

[B3] Aiga T. Manabe Y. Ito K. Chang T. C. Kabayama K. Ohshima S. (2020). Immunological evaluation of co-assembling a lipidated peptide antigen and lipophilic adjuvants: Self-adjuvanting anti-breast cancer vaccine candidates. Angew. Chem. 132 (40), 17705–17711. 10.1002/anie.202007999 32583549

[B4] Antonarelli G. Corti C. Tarantino P. Ascione L. Cortes J. Romero P. (2021). Therapeutic cancer vaccines revamping: Technology advancements and pitfalls. Ann. Oncol. 32 (12), 1537–1551. 10.1016/j.annonc.2021.08.2153 34500046 PMC8420263

[B5] Askoxylakis V. Zitzmann S. Mier W. Graham K. Krämer S. Von Wegner F. (2005). Preclinical evaluation of the breast cancer cell-binding peptide, p160. Clin. Cancer Res. 11 (18), 6705–6712. 10.1158/1078-0432.CCR-05-0432 16166451

[B6] Barati N. Nikpoor A. R. Mosaffa F. Razazan A. Badiee A. Motavallihaghi S. M. (2022). AE36 HER2/neu-derived peptide linked to positively charged liposomes with CpG-ODN as an effective therapeutic and prophylactic vaccine for breast cancer. J. Drug Deliv. Sci. Technol. 67, 102904. 10.1016/j.jddst.2021.102904

[B7] Beltran A. Graves L. Blancafort P. (2014). Novel role of Engrailed 1 as a prosurvival transcription factor in basal-like breast cancer and engineering of interference peptides block its oncogenic function. Oncogene 33 (39), 4767–4777. 10.1038/onc.2013.422 24141779 PMC4184217

[B8] Ben Djemaa S. David S. Hervé-Aubert K. Falanga A. Galdiero S. Allard-Vannier E. (2018). Formulation and *in vitro* evaluation of a siRNA delivery nanosystem decorated with gH625 peptide for triple negative breast cancer theranosis. Eur. J. Pharm. Biopharm. 131, 99–108. 10.1016/j.ejpb.2018.07.024 30063968

[B9] Ben Djemaa S. Hervé-Aubert K. Lajoie L. Falanga A. Galdiero S. Nedellec S. (2019). gH625 cell-penetrating peptide promotes the endosomal escape of nanovectorized siRNA in a triple-negative breast cancer cell line. Biomacromolecules 20 (8), 3076–3086. 10.1021/acs.biomac.9b00637 31305991

[B10] Bielenberg D. R. Pettaway C. A. Takashima S. Klagsbrun M. (2006). Neuropilins in neoplasms: Expression, regulation, and function. Exp. Cell Res. 312 (5), 584–593. 10.1016/j.yexcr.2005.11.024 16445911

[B11] Blanco E. Hsiao A. Mann A. P. Landry M. G. Meric‐Bernstam F. Ferrari M. (2011). Nanomedicine in cancer therapy: Innovative trends and prospects. Cancer Sci. 102 (7), 1247–1252. 10.1111/j.1349-7006.2011.01941.x 21447010 PMC11158341

[B12] Bolat Z. B. Nezir A. E. Devrim B. Zemheri E. Gulyuz S. Ozkose U. U. (2021). Delivery of doxorubicin loaded P18 conjugated-poly(2-ethyl-oxazoline)-DOPE nanoliposomes for targeted therapy of breast cancer. Toxicol. Appl. Pharmacol. 428, 115671. 10.1016/j.taap.2021.115671 34391753

[B13] Böhme D. Krieghoff J. Beck-Sickinger A. G. (2016). Double methotrexate-modified neuropeptide Y analogues express increased toxicity and overcome drug resistance in breast cancer cells. J. Med. Chem. 59 (7), 3409–3417. 10.1021/acs.jmedchem.6b00043 26985967

[B14] Browning L. M. Lee K. J. Cherukuri P. K. Huang T. Songkiatisak P. Warren S. (2018). Single gold nanoparticle plasmonic spectroscopy for study of chemical-dependent efflux function of single ABC transporters of single live Bacillus subtilis cells. Analyst 143 (7), 1599–1608. 10.1039/C7AN01787A 29488517 PMC5869163

[B15] Brunetti J. Depau L. Falciani C. Gentile M. Mandarini E. Riolo G. (2016). Insights into the role of sulfated glycans in cancer cell adhesion and migration through use of branched peptide probe. Sci. Rep. 6 (1), 27174–27213. 10.1038/srep27174 27255651 PMC4891694

[B16] Cabeza L. Ortiz R. Arias J. L. Prados J. Martínez M. A. R. Entrena J. M. (2015). Enhanced antitumor activity of doxorubicin in breast cancer through the use of poly (butylcyanoacrylate) nanoparticles. Int. J. Nanomedicine 10, 1291–1306. 10.2147/IJN.S74378 25709449 PMC4335619

[B17] Chan Y.-H. M. van Lengerich B. Boxer S. G. (2009). Effects of linker sequences on vesicle fusion mediated by lipid-anchored DNA oligonucleotides. Proc. Natl. Acad. Sci. 106 (4), 979–984. 10.1073/pnas.0812356106 19164559 PMC2633564

[B18] Chen R. Ni S. Chen W. Liu M. Feng J. Hu K. (2021). Improved anti-triple negative breast cancer effects of docetaxel by RGD-modified lipid-core micelles. Int. J. Nanomedicine 16, 5265–5279. 10.2147/IJN.S313166 34376979 PMC8349197

[B19] Chen X. Fan Z. Chen Y. Fang X. Sha X. (2013). Retro-inverso carbohydrate mimetic peptides with annexin1-binding selectivity, are stable *in vivo*, and target tumor vasculature. PLoS One 8 (12), e80390. 10.1371/journal.pone.0080390 24312470 PMC3846562

[B20] Cheng Y. Ji Y. (2019). RGD-modified polymer and liposome nanovehicles: Recent research progress for drug delivery in cancer therapeutics. Eur. J. Pharm. Sci. 128, 8–17. 10.1016/j.ejps.2018.11.023 30471410

[B21] Choi J. Yip-Schneider M. Albertin F. Wiesenauer C. Wang Y. Schmidt C. M. (2008). The effect of doxorubicin on MEK-ERK signaling predicts its efficacy in HCC. J. Surg. Res. 150 (2), 219–226. 10.1016/j.jss.2008.01.029 18468633

[B22] Claessens A. K. Ibragimova K. I. Geurts S. M. Bos M. E. Erdkamp F. L. Tjan-Heijnen V. C. (2020). The role of chemotherapy in treatment of advanced breast cancer: An overview for clinical practice. Crit. Rev. Oncology/Hematology 153, 102988. 10.1016/j.critrevonc.2020.102988 32599374

[B23] Corti C. Crimini E. Tarantino P. Pravettoni G. Eggermont A. M. Delaloge S. (2021). SARS-CoV-2 vaccines for cancer patients: A call to action. Eur. J. Cancer 148, 316–327. 10.1016/j.ejca.2021.01.046 33770576 PMC7904467

[B24] Corti C. Giachetti P. P. Eggermont A. M. Delaloge S. Curigliano G. (2022). Therapeutic vaccines for breast cancer: Has the time finally come? Eur. J. Cancer 160, 150–174. 10.1016/j.ejca.2021.10.027 34823982 PMC8608270

[B25] Cummings J. C. Zhang H. Jakymiw A. d'Avanzo N. Torrieri G. Figueiredo P. (2019). Peptide carriers to the rescue: Overcoming the barriers to siRNA delivery for cancer treatmentLinTT1 peptide-functionalized liposomes for targeted breast cancer therapy. Transl. Res. J. Pharm. 214597, 92120346–92121104. 10.1016/j.trsl.2019.07.01010.1016/j.ijpharm.2021.120346

[B187] d’Avanzo N. Torrieri G. Figueiredo P. Celia C. Paolino D. Correia A. (2021). LinTT1 peptide-functionalized liposomes for targeted breast cancer therapy. Int. J. Pharm. 597, 120346. 10.1016/j.ijpharm.2021.120346 33545283

[B26] Dafni U. Martin-Lluesma S. Balint K. Tsourti Z. Vervita K. Chenal J. (2021). Efficacy of cancer vaccines in selected gynaecological breast and ovarian cancers: A 20-year systematic review and meta-analysis. Eur. J. Cancer 142, 63–82. 10.1016/j.ejca.2020.10.014 33221598

[B27] Daniluk K. Kutwin M. Grodzik M. Wierzbicki M. Strojny B. Szczepaniak J. (2019). Use of selected carbon nanoparticles as melittin carriers for MCF-7 and MDA-MB-231 human breast cancer cells. Materials 13 (1), 90. 10.3390/ma13010090 31878020 PMC6981792

[B28] de Paula Peres L. da Luz F. A. C. dos Anjos Pultz B. Brigido P. C. de Araujo R. A. Goulart L. R. (2015). Peptide vaccines in breast cancer: The immunological basis for clinical response. Biotechnol. Adv. 33 (8), 1868–1877. 10.1016/j.biotechadv.2015.10.013 26523780

[B29] Deming T. J. (2016). Synthesis of side-chain modified polypeptides. Chem. Rev. 116 (3), 786–808. 10.1021/acs.chemrev.5b00292 26147333

[B30] Demircioglu F. Hodivala-Dilke K. (2016). αvβ3 integrin and tumour blood vessels-Learning from the past to shape the future. Curr. Opin. Cell Biol. 42, 121–127. 10.1016/j.ceb.2016.07.008 27474973

[B31] Depau L. Brunetti J. Falciani C. Scali S. Riolo G. Mandarini E. (2017). Coupling to a cancer-selective heparan-sulfate-targeted branched peptide can bypass breast cancer cell resistance to methotrexate. Oncotarget 8 (44), 76141–76152. 10.18632/oncotarget.19056 29100299 PMC5652693

[B32] DeSantis C. Ma J. Bryan L. Jemal A. (2014). Breast cancer statistics, 2013. CA a cancer J. Clin. 64 (1), 52–62. 10.3322/caac.21203 24114568

[B33] Dissanayake S. Denny W. A. Gamage S. Sarojini V. (2017). Recent developments in anticancer drug delivery using cell penetrating and tumor targeting peptides. J. Control. Release 250, 62–76. 10.1016/j.jconrel.2017.02.006 28167286

[B34] Du S. Luo C. Yang G. Gao H. Wang Y. Li X. (2020). Developing PEGylated reversed D-Peptide as a Novel HER2-targeted SPECT imaging probe for breast cancer detection. Bioconjugate Chem. 31 (8), 1971–1980. 10.1021/acs.bioconjchem.0c00334 32660241

[B35] Duffy C. Sorolla A. Wang E. Golden E. Woodward E. Davern K. (2020). Honeybee venom and melittin suppress growth factor receptor activation in HER2-enriched and triple-negative breast cancer. NPJ Precis. Oncol. 4 (1), 24–16. 10.1038/s41698-020-00129-0 32923684 PMC7463160

[B36] Eckstein F. (2014). Phosphorothioates, essential components of therapeutic oligonucleotides. Nucleic Acid. Ther. 24 (6), 374–387. 10.1089/nat.2014.0506 25353652

[B37] Fan L.-Q. Du G.-X. Li P.-F. Li M.-W. Sun Y. Zhao L.-M. (2016). Improved breast cancer cell-specific intracellular drug delivery and therapeutic efficacy by coupling decoration with cell penetrating peptide and SP90 peptide. Biomed. Pharmacother. 84, 1783–1791. 10.1016/j.biopha.2016.10.102 27899251

[B38] Fan L. Jin B. Zhang S. Song C. Li Q. (2016). Stimuli-free programmable drug release for combination chemo-therapy. Nanoscale 8 (25), 12553–12559. 10.1039/C5NR06305A 26554664

[B39] Faruk M. (2021). Breast cancer resistance to chemotherapy: When should we suspect it and how can we prevent it? Ann. Med. Surg. 70, 102793. 10.1016/j.amsu.2021.102793 PMC8519754 34691411

[B40] Feng G.-K. Liu R.-B. Zhang M.-Q. Ye X.-X. Zhong Q. Xia Y.-F. (2014). SPECT and near-infrared fluorescence imaging of breast cancer with a neuropilin-1-targeting peptide. J. Control. Release 192, 236–242. 10.1016/j.jconrel.2014.07.039 25058570

[B41] Fogal V. Zhang L. Krajewski S. Ruoslahti E. (2008). Mitochondrial/cell-surface protein p32/gC1qR as a molecular target in tumor cells and tumor stroma. Cancer Res. 68 (17), 7210–7218. 10.1158/0008-5472.CAN-07-6752 18757437 PMC2562323

[B42] Frasor J. Barnett D. H. Danes J. M. Hess R. Parlow A. F. Katzenellenbogen B. S. (2003). Response-specific and ligand dose-dependent modulation of estrogen receptor (ER) alpha activity by ERbeta in the uterus. Endocrinology 144 (7), 3159–3166. 10.1210/en.2002-0143 12810572

[B43] Fukunaga K. Tsutsumi H. Mihara H. (2019). Self-assembling peptides as building blocks of functional materials for biomedical applications. Bull. Chem. Soc. Jpn. 92 (2), 391–399. 10.1246/bcsj.20180293

[B44] Ganneau C. Simenel C. Emptas E. Courtiol T. Coïc Y.-M. Artaud C. (2017). Large-scale synthesis and structural analysis of a synthetic glycopeptide dendrimer as an anti-cancer vaccine candidate. Org. Biomol. Chem. 15 (1), 114–123. 10.1039/C6OB01931E 27812586

[B45] Garate A. Santos E. Pedraz J. L. Hernández R. M. Orive G. (2015). Evaluation of different RGD ligand densities in the development of cell-based drug delivery systems. J. Drug Target. 23 (9), 806–812. 10.3109/1061186X.2015.1020428 25816227

[B46] Genta I. Chiesa E. Colzani B. Modena T. Conti B. Dorati R. (2017). GE11 peptide as an active targeting agent in antitumor therapy: A minireview. Pharmaceutics 10 (1), 2. 10.3390/pharmaceutics10010002 29271876 PMC5874815

[B47] Gilbert A. Williams C. Azuero A. Burkard M. E. Kenzik K. Garrett-Mayer E. (2021). Utilizing data visualization to identify survival and treatment differences between women with de novo and recurrent metastatic breast cancer. Clin. Breast Cancer 21 (4), 292–301. 10.1016/j.clbc.2020.11.009 33309481

[B48] Gonzalez-Conchas G. A. Rodriguez-Romo L. Hernandez Barajas D. Gonzalez-Guerrero J. F. Rodriguez-Fernandez I. A. Verdines-Perez A. (2018). Epidermal growth factor receptor overexpression and outcomes in early breast cancer: A systematic review and a meta-analysis. Cancer Treat. Rev. 62, 1–8. 10.1016/j.ctrv.2017.10.008 29126017

[B49] Guidotti G. Brambilla L. Rossi D. (2017). Cell-penetrating peptides: From basic research to clinics. Trends Pharmacol. 38 (4), 406–424. 10.1016/j.tips.2017.01.003 28209404

[B50] Gupta K. P. Ward N. E. Gravitt K. R. Bergman P. J. O'Brian C. A. (1996). Partial reversal of multidrug resistance in human breast cancer cells by an N-myristoylated protein kinase C-α pseudosubstrate peptide (∗). J. Biol. Chem. 271 (4), 2102–2111. 10.1074/jbc.271.4.2102 8567666

[B51] Haggag Y. Abu Ras B. El-Tanani Y. Tambuwala M. M. McCarron P. Isreb M. (2020). Co-delivery of a RanGTP inhibitory peptide and doxorubicin using dual-loaded liposomal carriers to combat chemotherapeutic resistance in breast cancer cells. Expert Opin. Drug Deliv. 17 (11), 1655–1669. 10.1080/17425247.2020.1813714 32841584

[B52] Haggag Y. A. Matchett K. B. Falconer R. A. Isreb M. Jones J. Faheem A. (2019). Novel ran-RCC1 inhibitory peptide-loaded nanoparticles have anti-cancer efficacy *in vitro* and *in vivo* . Cancers 11 (2), 222. 10.3390/cancers11020222 30769871 PMC6406988

[B53] Hailing T. Yonghong P. Yufeng Z. Haitao T. (2022). Challenges for the application of EGFR-targeting peptide GE11 in tumor diagnosis and treatment. J. Control. Release 349, 592–605. 10.1016/j.jconrel.2022.07.018 35872181

[B54] Han W. Yuan Y. Li H. Fu Z. Wang M. Guan S. (2019). Design and anti-tumor activity of self-loaded nanocarriers of siRNA. Colloids Surfaces B Biointerfaces. 183, 110385. 10.1016/j.colsurfb.2019.110385 31408781

[B55] Hart C. D. Migliaccio I. Malorni L. Guarducci C. Biganzoli L. Di Leo A. (2015). Challenges in the management of advanced, ER-positive, HER2-negative breast cancer. Nat. Rev. Clin. Oncol. 12 (9), 541–552. 10.1038/nrclinonc.2015.99 26011489

[B56] Hatakeyama S. Sugihara K. Shibata T. K. Nakayama J. Akama T. O. Tamura N. (2011). Targeted drug delivery to tumor vasculature by a carbohydrate mimetic peptide. Proc. Natl. Acad. Sci. 108 (49), 19587–19592. 10.1073/PNAS.1105057108 22114188 PMC3241764

[B57] Hazeri Y. Samie A. Ramezani M. Alibolandi M. Yaghoobi E. Dehghani S. (2022). Dual-targeted delivery of doxorubicin by mesoporous silica nanoparticle coated with AS1411 aptamer and RGDK-R peptide to breast cancer *in vitro* and *in vivo* . J. Drug Deliv. Sci. Technol. 71, 103285. 10.1016/j.jddst.2022.103285

[B58] He C. Lu K. Liu D. Lin W. (2014). Nanoscale metal–organic frameworks for the co-delivery of cisplatin and pooled siRNAs to enhance therapeutic efficacy in drug-resistant ovarian cancer cells. J. Am. Chem. Soc. 136 (14), 5181–5184. 10.1021/ja4098862 24669930 PMC4210117

[B59] Helguero L. A. Faulds M. H. Gustafsson J.-Å. Haldosen L.-A. (2005). Estrogen receptors alfa (ERalpha) and beta (ERbeta) differentially regulate proliferation and apoptosis of the normal murine mammary epithelial cell line HC11. Oncogene 24 (44), 6605–6616. 10.1038/SJ.ONC.1208807 16007178

[B60] Herbst R. S. Shin D. M. (2002). Monoclonal antibodies to target epidermal growth factor receptor-positive tumors: A new paradigm for cancer therapy. Cancer 94 (5), 1593–1611. 10.1002/cncr.10372 11920518

[B61] Holst F. Stahl P. R. Ruiz C. Hellwinkel O. Jehan Z. Wendland M. (2007). Estrogen receptor alpha (ESR1) gene amplification is frequent in breast cancer. Nat. Genet. 39 (5), 655–660. 10.1038/ng2006 17417639

[B62] Honarvar H. Calce E. Doti N. Langella E. Orlova A. Buijs J. (2018). Evaluation of HER2-specific peptide ligand for its employment as radiolabeled imaging probe. Sci. Rep. 8 (1), 2998–3012. 10.1038/s41598-018-21283-3 29445216 PMC5812989

[B63] Hou G. Qian J. Xu W. Sun T. Wang J. Wang Y. (2019). Multifunctional PEG-b-polypeptide-decorated gold nanorod for targeted combined chemo-photothermal therapy of breast cancer. Colloids Surfaces B Biointerfaces. 181, 602–611. 10.1016/j.colsurfb.2019.05.025 31202131

[B64] Hou J. Deng M. Li X. Liu W. Chu X. Wang J. (2015). Chaperone gp96 mediates ER-α36 cell membrane expression. Oncotarget 6 (31), 31857–31867. 10.18632/oncotarget.5273 26396174 PMC4741645

[B65] Hou J. Li X. Li C. Sun L. Zhao Y. Zhao J. (2015). Plasma membrane gp96 enhances invasion and metastatic potential of liver cancer via regulation of uPAR. Mol. Oncol. 9 (7), 1312–1323. 10.1016/j.molonc.2015.03.004 25841765 PMC5528822

[B66] Ishida T. Tsujisaki M. Hinoda Y. Imai K. Yachi A. (1994). Establishment and characterization of mouse-human chimeric monoclonal antibody to erbB-2 product. Jpn. J. Cancer Res. 85 (2), 172–178. 10.1111/j.1349-7006.1994.tb02079.x 7908285 PMC5919429

[B67] Jacobson H. I. Andersen T. T. Bennett J. A. (2014). Development of an active site peptide analog of α-fetoprotein that prevents breast cancer. Cancer Prev. Res. 7 (6), 565–573. 10.1158/1940-6207.CAPR-13-0405 24706695

[B68] Jain V. Kumar H. Anod H. V. Chand P. Gupta N. V. Dey S. (2020). A review of nanotechnology-based approaches for breast cancer and triple-negative breast cancer. J. Control. Release 326, 628–647. 10.1016/j.jconrel.2020.07.003 32653502

[B69] Jamdade V. S. Sethi N. Mundhe N. A. Kumar P. Lahkar M. Sinha N. (2015). Therapeutic targets of triple-negative breast cancer: A review. Br. J. Pharmacol. 172 (17), 4228–4237. 10.1111/bph.13211 26040571 PMC4556464

[B70] Jin J.-O. Kim G. Hwang J. Han K. H. Kwak M. Lee P. C. (2020). Nucleic acid nanotechnology for cancer treatment. Biochimica Biophysica Acta (BBA)-Reviews Cancer 1874 (1), 188377. 10.1016/j.bbcan.2020.188377 32418899

[B71] Kim B. Shin J. Wu J. Omstead D. T. Kiziltepe T. Littlepage L. E. (2020). Engineering peptide-targeted liposomal nanoparticles optimized for improved selectivity for HER2-positive breast cancer cells to achieve enhanced *in vivo* efficacy. J. Control. Release 322, 530–541. 10.1016/j.jconrel.2020.04.010 32276005 PMC7932755

[B72] Kim J.-W. Akiyama M. Park J.-H. Lin M.-L. Shimo A. Ueki T. (2009). Activation of an estrogen/estrogen receptor signaling by BIG3 through its inhibitory effect on nuclear transport of PHB2/REA in breast cancer. Nat. Preced. 1, 1468–1478. 10.1111/j.1349-7006.2009.01209.x PMC11159637 19496786

[B73] Kim W.-J. Koo J.-H. Cho H.-J. Lee J.-U. Kim J. Y. Lee H.-G. (2018). Protein tyrosine phosphatase conjugated with a novel transdermal delivery peptide, astrotactin 1–derived peptide recombinant protein tyrosine phosphatase (AP-rPTP), alleviates both atopic dermatitis–like and psoriasis-like dermatitis. J. Allergy Clin. Immunol. 141 (1), 137–151. 10.1016/j.jaci.2017.04.007 28456618

[B74] Kuai Q. Wang Y. Gao F. Qi Y. Wang R. Wang Y. (2019). Peptide self-assembly nanoparticles loaded with panobinostat to activate latent human immunodeficiency virus. J. Biomed. Nanotechnol. 15 (5), 979–992. 10.1166/jbn.2019.2764 30890229

[B75] Kumar S. R. Deutscher S. L. (2008). 111In-labeled galectin-3 targeting peptide as a SPECT agent for imaging breast tumors. J. Nucl. Med. 49 (5), 796–803. 10.2967/jnumed.107.048751 18413389

[B76] Larimer B. M. Deutscher S. L. (2014). Development of a peptide by phage display for SPECT imaging of resistance-susceptible breast cancer. Am. J. Nucl. Med. Mol. 4 (5), 435–447. 10.1002/aja.1000530305 PMC4138138 25143862

[B77] Larimer B. M. Thomas W. D. Smith G. P. Deutscher S. L. (2014). Affinity maturation of an ERBB2-targeted SPECT imaging peptide by *in vivo* phage display. Mol. Imaging Biol. 16 (4), 449–458. 10.1007/s11307-014-0724-5 24550054

[B78] Li J. Liu F. Shao Q. Min Y. Costa M. Yeow E. K. (2014). Enzyme-responsive cell-penetrating peptide conjugated mesoporous silica quantum dot nanocarriers for controlled release of nucleus targeted drug molecules and real time intracellular fluorescence imaging of tumor cells. Adv. Healthc. Mater. 3 (8), 1230–1239. 10.1002/adhm.201300613 24550203

[B79] Li K. C. Pang L. Y. Pan X. R. Fan S. N. Wang X. X. Wang Q. Y. (2021). GE11 modified PLGA/TPGS nanoparticles targeting delivery of salinomycin to breast cancer cells. Technol. Cancer Res. Treat. 20, 15330338211004954–15330338211004957. 10.1177/15330338211004954 34056977 PMC8182624

[B80] Li W. Li H. Zhang L. Hu M. Li F. Deng J. (2017). Long non-coding RNA LINC00672 contributes to p53 protein-mediated gene suppression and promotes endometrial cancer chemosensitivity. J. Biol. Chem. 292 (14), 5801–5813. 10.1074/jbc.M116.758508 28232485 PMC5392574

[B81] Li W. Nicol F. Szoka F. C. Jr (2004). Gala: A designed synthetic pH-responsive amphipathic peptide with applications in drug and gene delivery. Adv. Drug Deliv. Rev. 56 (7), 967–985. 10.1016/j.addr.2003.10.041 15066755

[B82] Li X. Wang B. Liu W. Gui M. Peng Z. Meng S. (2015). Blockage of conformational changes of heat shock protein gp96 on cell membrane by a α-helix peptide inhibits HER2 dimerization and signaling in breast cancer. PLoS One 10 (4), e0124647. 10.1371/journal.pone.0124647 25898135 PMC4405268

[B83] Li Y. Clark K. A. Tan Z. (2018). Methods for engineering therapeutic peptides. Chin. Chem. Lett. 29 (7), 1074–1078. 10.1016/j.cclet.2018.05.027

[B84] Liang Z. Du L. Zhang E. Zhao Y. Wang W. Ma P. (2021). Targeted-delivery of siRNA via a polypeptide-modified liposome for the treatment of gp96 over-expressed breast cancer. Mater. Sci. Eng. C 121, 111847. 10.1016/j.msec.2020.111847 33579510

[B85] Lingeman R. G. Hickey R. J. Malkas L. H. (2014). Expression of a novel peptide derived from PCNA damages DNA and reverses cisplatin resistance. Cancer Chemother. Pharmacol. 74 (5), 981–993. 10.1007/s00280-014-2574-x 25190177 PMC5458613

[B86] Liu J. Guo N. Gao C. Liu N. Zheng X. Tan Y. (2019). Effective gene silencing mediated by polypeptide nanoparticles LAH4-L1-siMDR1 in multi-drug resistant human breast cancer. J. Biomed. Nanotechnol. 15 (3), 531–543. 10.1166/jbn.2019.2705 31165698

[B87] Liu M. Wang H. Liu L. Wang B. Sun G. (2016). Melittin-MIL-2 fusion protein as a candidate for cancer immunotherapy. J. Transl. Med. 14 (1), 155–212. 10.1186/s12967-016-0910-0 27246873 PMC4888606

[B88] Loftus P. G. Watson L. Deedigan L. M. Camarillo-Retamosa E. Dwyer R. M. O'Flynn L. (2021). Targeting stromal cell Syndecan-2 reduces breast tumour growth, metastasis and limits immune evasion. Int. J. Cancer 148 (5), 1245–1259. 10.1002/ijc.33383 33152121 PMC7839764

[B89] Ma F. Wu J. Fu L. Li A. Lan B. Chen K. (2021). Interpretation of specification for breast cancer screening, early diagnosis, and treatment management in Chinese women. J. Natl. Cancer Cent. 1 (3), 97–100. 10.1016/j.jncc.2021.07.003 PMC11256670 39036376

[B90] Maeda H. (2015). Toward a full understanding of the EPR effect in primary and metastatic tumors as well as issues related to its heterogeneity. Adv. Drug Deliv. Rev. 91, 3–6. 10.1016/j.addr.2015.01.002 25579058

[B91] Mahdavi M. Moreau V. (2016). *In silico* designing breast cancer peptide vaccine for binding to MHC class I and II: A molecular docking study. Comput. Biol. Chem. 65, 110–116. 10.1016/j.compbiolchem.2016.10.007 27816827

[B92] Mahdavi M. Moreau V. Kheirollahi M. (2017). Identification of B and T cell epitope based peptide vaccine from IGF-1 receptor in breast cancer. J. Mol. Graph. Model. 75, 316–321. 10.1016/j.jmgm.2017.06.004 28628857

[B93] Mansouri W. Fordyce S. B. Wu M. Jones D. Cohn D. Lin Q. (2018). Efficacy and tolerability of AFPep, a cyclic peptide with anti-breast cancer properties. Toxicol. Appl. Pharmacol. 345, 10–18. 10.1016/j.taap.2018.03.004 29518411

[B94] Marra A. Viale G. Curigliano G. (2019). Recent advances in triple negative breast cancer: The immunotherapy era. BMC Med. 17 (1), 90–99. 10.1186/s12916-019-1326-5 31068190 PMC6507064

[B95] Mazo A. R. Allison-Logan S. Karimi F. Chan N. J.-A. Qiu W. Duan W. (2020). Ring opening polymerization of α-amino acids: Advances in synthesis, architecture and applications of polypeptides and their hybrids. Chem. Soc. Rev. 49 (14), 4737–4834. 10.1039/C9CS00738E 32573586

[B96] Mazumder A. Shiao S. Haricharan S. (2021). HER2 activation and endocrine treatment resistance in HER2-negative breast cancer. Endocrinology 162 (10), bqab153. 10.1210/endocr/bqab153 34320193 PMC8379900

[B97] Michalska M. Florczak A. Dams-Kozlowska H. Gapinski J. Jurga S. Schneider R. (2016). Peptide-functionalized ZCIS QDs as fluorescent nanoprobe for targeted HER2-positive breast cancer cells imaging. Acta Biomater. 35, 293–304. 10.1016/j.actbio.2016.02.002 26850146

[B98] Milletti F. (2012). Cell-penetrating peptides: Classes, origin, and current landscape. Drug Discov. Today 17 (15-16), 850–860. 10.1016/j.drudis.2012.03.002 22465171

[B99] Miyako H. Kametani Y. Katano I. Ito R. Tsuda B. Furukawa A. (2011). Antitumor effect of new HER2 peptide vaccination based on B cell epitope. Anticancer Res. 31 (10), 3361–3368. 10.1245/s10434-011-2001-z 21965747

[B100] Morris S. Ahmad N. André S. Kaltner H. Gabius H.-J. Brenowitz M. (2004). Quaternary solution structures of galectins-1,-3, and-7. Glycobiology 14 (3), 293–300. 10.1093/glycob/cwh029 14693909

[B101] Motiei M. Aboutalebi F. Forouzanfar M. Dormiani K. Nasr-Esfahani M. H. Mirahmadi-Zare S. Z. (2021). Smart co-delivery of miR-34a and cytotoxic peptides (LTX-315 and melittin) by chitosan based polyelectrolyte nanocarriers for specific cancer cell death induction. Mater. Sci. Eng. C 128, 112258. 10.1016/j.msec.2021.112258 34474818

[B102] Mozaffari S. Salehi D. Mahdipoor P. Beuttler R. Tiwari R. Aliabadi H. M. (2021). Design and application of hybrid cyclic-linear peptide-doxorubicin conjugates as a strategy to overcome doxorubicin resistance and toxicity. Eur. J. Med. Chem. 226, 113836. 10.1016/j.ejmech.2021.113836 34537446

[B103] Nam S. H. Jang J. Cheon D. H. Chong S.-E. Ahn J. H. Hyun S. (2021). pH-Activatable cell penetrating peptide dimers for potent delivery of anticancer drug to triple-negative breast cancer. J. Control. Release 330, 898–906. 10.1016/j.jconrel.2020.10.063 33152392

[B104] Nieberler M. Reuning U. Reichart F. Notni J. Wester H.-J. Schwaiger M. (2017). Exploring the role of RGD-recognizing integrins in cancer. Cancers 9 (9), 116. 10.3390/cancers9090116 28869579 PMC5615331

[B105] Nilsson S. Koehler K. F. Gustafsson J.-Å. (2011). Development of subtype-selective oestrogen receptor-based therapeutics. Nat. Rev. Drug Discov. 10 (10), 778–792. 10.1038/nrd3551 21921919

[B106] Numata K. (2015). Poly (amino acid)s/polypeptides as potential functional and structural materials. Polym. J. 47 (8), 537–545. 10.1038/pj.2015.35

[B107] Okines A. F. Cunningham D. (2010). Trastuzumab in gastric cancer. Eur. J. Cancer 46 (11), 1949–1959. 10.1016/j.ejca.2010.05.003 20542421

[B108] Orr G. A. Han E. Browne P. Nieves E. O'Connor B. Yang C. (1993). Identification of the major phosphorylation domain of murine mdr1b P-glycoprotein.Analysis of the protein kinase A and protein kinase C phosphorylation sites. J. Biol. Chem. 268 (33), 25054–25062. 10.1016/s0021-9258(19)74570-0 7901220

[B109] Pan G. Jin X. Mou Q. Zhang C. (2017). Recent progress on DNA block copolymer. Chin. Chem. Lett. 28 (9), 1822–1828. 10.1016/j.cclet.2017.08.022

[B110] Parikh A. B. Kozuch P. Rohs N. Becker D. J. Levy B. P. (2017). Metformin as a repurposed therapy in advanced non-small cell lung cancer (NSCLC): Results of a phase II trial. Investig. New Drugs 35 (6), 813–819. 10.1007/s10637-017-0511-7 28936567

[B111] Parise C. A. Caggiano V. (2014). Breast cancer survival defined by the ER/PR/HER2 subtypes and a surrogate classification according to tumor grade and immunohistochemical biomarkers. J. Cancer Epidemiol. 2014 (1), 469251–469311. 10.1155/2014/469251 24955090 PMC4058253

[B112] Park S. E. El-Sayed N. S. Shamloo K. Lohan S. Kumar S. Sajid M. I. (2021). Targeted delivery of cabazitaxel using cyclic cell-penetrating peptide and biomarkers of extracellular matrix for prostate and breast cancer therapy. Bioconjugate Chem. 32 (8), 1898–1914. 10.1021/acs.bioconjchem.1c00319 34309357

[B113] Parker K. L. Schimmer B. P. (2001). “Pituitary hormones and their hypothalamic releasing factors,” in Goodman and gilman’s the pharmacological basis of therapeutics Editors Hardman J. G. Limbird L. E. Goodman Gilman A. (New York: McGraw-Hill), 1541–1562.

[B114] Pérez-Herrero E. Fernández-Medarde A. (2015). Advanced targeted therapies in cancer: Drug nanocarriers, the future of chemotherapy. Eur. J. Pharm. Biopharm. 93, 52–79. 10.1016/j.ejpb.2015.03.018 25813885

[B115] Pescina S. Ostacolo C. Gomez-Monterrey I. Sala M. Bertamino A. Sonvico F. (2018). Cell penetrating peptides in ocular drug delivery: State of the art. J. Control. Release 284, 84–102. 10.1016/j.jconrel.2018.06.023 29913221

[B116] Pierschbacher M. D. Ruoslahti E. (1984). Cell attachment activity of fibronectin can be duplicated by small synthetic fragments of the molecule. Nature 309 (5963), 30–33. 10.1038/309030a0 6325925

[B117] Qian L. Fan H. Ju Y. Chen L. Li X. Ye X. (2019). A peptide-based inhibitor of gp96 suppresses HBsAg expression and HBV replication by upregulation of p53. J. General Virology 100 (8), 1241–1252. 10.1099/jgv.0.001289 31204972

[B118] Qin W. Xie M. Qin X. Fang Q. Yin F. Li Z. (2018). Recent advances in peptidomimetics antagonists targeting estrogen receptor α-coactivator interaction in cancer therapy. Bioorg. Med. Chem. Lett. 28 (17), 2827–2836. 10.1016/j.bmcl.2018.05.062 30025900

[B119] Qiubo W. Yingnian L. Yunjuan Z. Yanli Z. (2000). Study on the immune-regulating mechanism of the bee venom. Zhongguo Mian yi xue za zhi= Chin. J. Immunol. 16 (10), 542–544. 10.3321/j.issn:1000-484X.2000.10.008

[B120] Raveendran R. Chen F. Kent B. Stenzel M. H. (2020). Estrone-decorated polyion complex micelles for targeted melittin delivery to hormone-responsive breast cancer cells. Biomacromolecules 21 (3), 1222–1233. 10.1021/acs.biomac.9b01681 32022540

[B121] Reches M. Gazit E. (2003). Casting metal nanowires within discrete self-assembled peptide nanotubes. Science 300 (5619), 625–627. 10.1126/science.1082387 12714741

[B122] Reissmann S. (2014). Cell penetration: Scope and limitations by the application of cell penetrating peptides. J. Peptide Sci. 20 (10), 760–784. 10.1002/psc.2672 25112216

[B123] Riis M. (2020). Modern surgical treatment of breast cancer. Ann. Med. Surg. 56, 95–107. 10.1016/j.amsu.2020.06.016 PMC7327379 32637082

[B124] Rubinstein D. B. Stortchevoi A. Boosalis M. Ashfaq R. Ghebrehiwet B. Peerschke E. I. (2004). Receptor for the globular heads of C1q (gC1q-R, p33, hyaluronan-binding protein) is preferentially expressed by adenocarcinoma cells. Int. J. Cancer 110 (5), 741–750. 10.1002/ijc.20105 15146564

[B125] Schmid H A. (2008). Pasireotide (SOM230): Development, mechanism of action and potential applications. Mol. Cell. Endocrinol. 286 (1-2), 69–74. 10.1016/j.mce.2007.09.006 17977644

[B126] Schrama D. Reisfeld R. A. Becker J. C. (2006). Antibody targeted drugs as cancer therapeutics. Nat. Rev. DrugDiscovery 5 (2), 147–159. 10.1038/nrd1957 16424916

[B127] Seelig A. Gatlik-Landwojtowicz E. (2005). Inhibitors of multidrug efflux transporters: Their membrane and protein interactions. Mini Rev. Med. Chem. 5 (2), 135–151. 10.2174/1389557053402693 15720284

[B128] Servín-Blanco R. Chávaro-Ortiz R. M. Zamora-Alvarado R. Martínez-Cortes F. Gevorkian G. Manoutcharian K. (2018). Generation of cancer vaccine immunogens derived from major histocompatibility complex (MHC) class I molecules using variable epitope libraries. Immunol. Lett. 204, 47–54. 10.1016/j.imlet.2018.10.008 30339819

[B129] Shahin M. Soudy R. Aliabadi H. M. Kneteman N. Kaur K. Lavasanifar A. (2013). Engineered breast tumor targeting peptide ligand modified liposomal doxorubicin and the effect of peptide density on anticancer activity. Biomaterials 34 (16), 4089–4097. 10.1016/j.biomaterials.2013.02.019 23465829

[B130] Sharma S. Kotamraju V. R. Molder T. Tobi A. Teesalu T. Ruoslahti E. (2017). Tumor-penetrating nanosystem strongly suppresses breast tumor growth. Nano Lett. 17 (3), 1356–1364. 10.1021/acs.nanolett.6b03815 28178415 PMC5819594

[B131] Sheng Y. You Y. Chen Y. (2016). Dual-targeting hybrid peptide-conjugated doxorubicin for drug resistance reversal in breast cancer. Int. J. Pharm. 512 (1), 1–13. 10.1016/j.ijpharm.2016.08.016 27521706

[B132] Shi N.-Q. Qi X.-R. Xiang B. Zhang Y. (2014). A survey on “trojan horse” peptides: Opportunities, issues and controlled entry to “troy”. J. Control. Release 194, 53–70. 10.1016/j.jconrel.2014.08.014 25151981

[B133] Shukla R. S. Qin B. Cheng K. (2014). Peptides used in the delivery of small noncoding RNA. Mol. Pharm. 11 (10), 3395–3408. 10.1021/mp500426r 25157701 PMC4186677

[B134] Siegel R. L. Miller K. D. Jemal A. (2020). Cancer statistics, 2020. CA A Cancer J. Clin. 70 (1), 7–30. 10.3322/caac.21590 31912902

[B135] Simón-Gracia L. Hunt H. Teesalu T. (2018). Peritoneal carcinomatosis targeting with tumor homing peptides. Molecules 23 (5), 1190. 10.3390/molecules23051190 29772690 PMC6100015

[B136] Simón-Gracia L. Scodeller P. Fuentes S. S. Vallejo V. G. Ríos X. San Sebastián E. (2018). Application of polymersomes engineered to target p32 protein for detection of small breast tumors in mice. Oncotarget 9 (27), 18682–18697. 10.18632/oncotarget.24588 29721153 PMC5922347

[B137] Singh T. Murthy A. S. Yang H.-J. Im J. (2018). Versatility of cell-penetrating peptides for intracellular delivery of siRNA. Drug Deliv. 25 (1), 1996–2006. 10.1080/10717544.2018.1543366 30799658 PMC6319457

[B138] Slamon D. J. Clark G. M. Wong S. G. Levin W. J. Ullrich A. McGuire W. L. (1987). Human breast cancer: Correlation of relapse and survival with amplification of the HER-2/neu oncogene. Science 235 (4785), 177–182. 10.1126/science.3798106 3798106

[B139] Sobhani N. Scaggiante B. Morris R. Chai D. Catalano M. Tardiel-Cyril D. R. (2022). Therapeutic cancer vaccines: From biological mechanisms and engineering to ongoing clinical trials. Cancer Treat. Rev. 109, 102429. 10.1016/j.ctrv.2022.102429 35759856 PMC9217071

[B140] Song Y. Challa S. R. Medforth C. J. Qiu Y. Watt R. K. Peña D. (2004). Synthesis of peptide-nanotube platinum-nanoparticle composites. Chem. Commun. 9, 1044–1045. 10.1039/B402126F 15116176

[B141] Sorolla A. Ho D. Wang E. Evans C. Ormonde C. Rashwan R. (2016). Sensitizing basal-like breast cancer to chemotherapy using nanoparticles conjugated with interference peptide. Nanoscale 8 (17), 9343–9353. 10.1039/C5NR08331A 27089946

[B142] Sorolla A. Sorolla M. A. Wang E. Ceña V. (2020). Peptides, proteins and nanotechnology: A promising synergy for breast cancer targeting and treatment. Expert Opin. Drug Deliv. 17 (11), 1597–1613. 10.1080/17425247.2020.1814733 32835538

[B143] Sorolla A. Wang E. Clemons T. D. Evans C. W. Plani-Lam J. H. Golden E. (2019). Triple-hit therapeutic approach for triple negative breast cancers using docetaxel nanoparticles, EN1-iPeps and RGD peptides. Nanomedicine Nanotechnol. Biol. Med. 20, 102003. 10.1016/j.nano.2019.04.006 31055077

[B144] Sorolla A. Wang E. Golden E. Duffy C. Henriques S. T. Redfern A. D. (2020). Precision medicine by designer interference peptides: Applications in oncology and molecular therapeutics. Oncogene 39 (6), 1167–1184. 10.1038/s41388-019-1056-3 31636382 PMC7002299

[B145] Stefanick J. F. Kiziltepe T. Bilgicer B. (2015). Improved peptide-targeted liposome design through optimized peptide hydrophilicity, ethylene glycol linker length, and peptide density. J. Biomed. Nanotechnol. 11 (8), 1418–1430. 10.1166/jbn.2015.2087 26295142

[B146] Strzalka W. Ziemienowicz A. (2011). Proliferating cell nuclear antigen (PCNA): A key factor in DNA replication and cell cycle regulation. Ann. Bot. 107 (7), 1127–1140. 10.1093/aob/mcq243 21169293 PMC3091797

[B147] Subbarao N. K. Parente R. A. Szoka F. C. Jr Nadasdi L. Pongracz K. (1987). pH-dependent bilayer destabilization by an amphipathic peptide. Biochemistry 26 (11), 2964–2972. 10.1021/bi00385a002 2886149

[B148] Suo A. Qian J. Xu M. Xu W. Zhang Y. Yao Y. (2017). Folate-decorated PEGylated triblock copolymer as a pH/reduction dual-responsive nanovehicle for targeted intracellular co-delivery of doxorubicin and Bcl-2 siRNA. Mater. Sci. Eng. C 76, 659–672. 10.1016/j.msec.2017.03.124 28482576

[B149] Suo A. Qian J. Zhang Y. Liu R. Xu W. Wang H. (2016). Comb-like amphiphilic polypeptide-based copolymer nanomicelles for co-delivery of doxorubicin and P-gp siRNA into MCF-7 cells. Mater. Sci. Eng. C 62, 564–573. 10.1016/j.msec.2016.02.007 26952460

[B150] Tai W. Gao X. (2017). Functional peptides for siRNA delivery. Adv. Drug Deliv. Rev. 110, 157–168. 10.1016/j.addr.2016.08.004 27530388 PMC5305781

[B151] Talesh G. A. Ebrahimi Z. Badiee A. Mansourian M. Attar H. Arabi L. (2016). Poly (I: C)-DOTAP cationic nanoliposome containing multi-epitope HER2-derived peptide promotes vaccine-elicited anti-tumor immunity in a murine model. Immunol. Lett. 176, 57–64. 10.1016/j.imlet.2016.05.016 27260485

[B152] Terjung A. Kummer S. Friedrich M. (2014). Simultaneous 24 h-infusion of high-dose 5-fluorouracil and sodium-folinate as alternative to capecitabine in advanced breast cancer. Anticancer Res. 34 (12), 7233–7238. 25503154

[B153] Terwilliger C. Eisenberg D. (1982). The structure of melittin. J. Biol. Chem. 257, 6–016. 7076662

[B154] Tesauro D. Accardo A. Diaferia C. Milano V. Guillon J. Ronga L. (2019). Peptide-based drug-delivery systems in biotechnological applications: Recent advances and perspectives. Molecules 24 (2), 351. 10.3390/molecules24020351 30669445 PMC6359574

[B155] Vreeland T. J. Litton J. K. Qiao N. Philips A. V. Alatrash G. Hale D. F. (2018). Phase ib trial of folate binding protein (FBP)-derived peptide vaccines, E39 and an attenuated version, E39’: An analysis of safety and immune response. Clin. Immunol. 192, 6–13. 10.1016/j.clim.2018.03.010 29574039 PMC5988975

[B156] Wallis J. Katti P. Martin A. M. Hills T. Seymour L. W. Shenton D. P. (2020). A liposome-based cancer vaccine for a rapid and high-titre anti-ErbB-2 antibody response. Eur. J. Pharm. Sci. 152, 105456. 10.1016/j.ejps.2020.105456 32653563

[B157] Wang H. Wang X. Zhang Y. Cheng R. Yuan J. Zhong Z. (2020). Systemic delivery of NAC-1 siRNA by neuropilin-targeted polymersomes sensitizes antiangiogenic therapy of metastatic triple-negative breast cancer. Biomacromolecules 21 (12), 5119–5127. 10.1021/acs.biomac.0c01253 33174734

[B158] Wang J. Li N. Cao L. Gao C. Zhang Y. Shuai Q. (2020). DOX-loaded peptide dendritic copolymer nanoparticles for combating multidrug resistance by regulating the lysosomal pathway of apoptosis in breast cancer cells. J. Mater. Chem. B 8 (6), 1157–1170. 10.1039/C9TB02130B 31951231

[B159] Wang R. Shen Q. Li X. Xie C. Lu W. Wang S. (2018). Efficacy of inverso isomer of CendR peptide on tumor tissue penetration. Acta Pharm. Sin. B 8 (5), 825–832. 10.1016/j.apsb.2018.06.006 30245969 PMC6146380

[B160] Wang W. Li Y. Wang Y. Ren S. Li Y. Wang B. (2018). Polyactin A is a novel and potent immunological adjuvant for peptide-based cancer vaccine. Int. Immunopharmacol. 54, 95–102. 10.1016/j.intimp.2017.10.020 29112895

[B161] Wang Y. Wu S. Zhu X. Zhang L. Deng J. Li F. (2020). LncRNA-encoded polypeptide ASRPS inhibits triple-negative breast cancer angiogenesis. J. Exp. Med. 217 (3), jem.20190950. 10.1084/jem.20190950 31816634 PMC7062514

[B162] Wang Y. Yin S. Mei L. Yang Y. Xu S. He X. (2020). A dual receptors-targeting and size-switchable “cluster bomb” co-loading chemotherapeutic and transient receptor potential ankyrin 1 (TRPA-1) inhibitor for treatment of triple negative breast cancer. J. Control. Release 321, 71–83. 10.1016/j.jconrel.2020.02.010 32035191

[B163] Watson D. S. Endsley A. N. Huang L. (2012). Design considerations for liposomal vaccines: Influence of formulation parameters on antibody and cell-mediated immune responses to liposome associated antigens. Vaccine 30 (13), 2256–2272. 10.1016/j.vaccine.2012.01.070 22306376 PMC3296885

[B164] Wilson T. Longley D. Johnston P. (2006). Chemoresistance in solid tumours. Ann. Oncol. 17, 315–324. 10.1093/annonc/mdl280 17018746

[B165] Wu P.-H. Opadele A. E. Onodera Y. Nam J.-M. (2019). Targeting integrins in cancer nanomedicine: Applications in cancer diagnosis and therapy. Cancers 11 (11), 1783. 10.3390/cancers11111783 31766201 PMC6895796

[B166] Xu J. Khan A. R. Fu M. Wang R. Ji J. Zhai G. (2019). Cell-penetrating peptide: A means of breaking through the physiological barriers of different tissues and organs. J. Control. Release 309, 106–124. 10.1016/j.jconrel.2019.07.020 31323244

[B167] Yang S. Leong J. Wang Y. Sim R. Tan K. H. Chua Y. H. (2022). Drug-free neutrally charged polypeptide nanoparticles as anticancer agents. J. Control. Release 345, 464–474. 10.1016/j.jconrel.2022.03.034 35331785

[B168] Yang Y. Jia Y. Xiao Y. Hao Y. Zhang L. Chen X. (2018). Tumor‐targeting anti‐microRNA‐155 delivery based on biodegradable poly (ester amine) and hyaluronic acid shielding for lung cancer therapy. ChemPhysChem 19 (16), 2058–2069. 10.1002/cphc.201701375 29488305

[B169] Yoshimaru T. Komatsu M. Matsuo T. Chen Y.-A. Murakami Y. Mizuguchi K. (2013). Targeting BIG3–PHB2 interaction to overcome tamoxifen resistance in breast cancer cells. Nat. Commun. 4 (1), 2443–2455. 10.1038/ncomms3443 24051437 PMC3791465

[B170] Yu D.-H. Liu Y.-R. Luan X. Liu H.-J. Gao Y.-G. Wu H. (2015). IF7-conjugated nanoparticles target Annexin 1 of tumor vasculature against P-gp mediated multidrug resistance. Bioconjugate Chem. 26 (8), 1702–1712. 10.1021/acs.bioconjchem.5b00283 26076081

[B171] Yuan Y. Wang L. Du W. Ding Z. Zhang J. Han T. (2015). Intracellular self-assembly of Taxol nanoparticles for overcoming multidrug resistance. Angew. Chem. Int. Ed. 54 (33), 9700–9704. 10.1002/anie.201504329 26118539

[B172] Zamani P. Mashreghi M. Bazaz M. R. Mirzavi F. Barati M. Zahedipour F. (2022). Improving potency of nanoliposomal AE36 peptide vaccine by adding CD4+ T cell helper epitope and MPL in TUBO breast cancer mice model. J. Drug Deliv. Sci. Technol. 71, 103346. 10.1016/j.jddst.2022.103346

[B173] Zamani P. Navashenaq J. G. Nikpoor A. R. Hatamipour M. Oskuee R. K. Badiee A. (2019). MPL nano-liposomal vaccine containing P5 HER2/neu-derived peptide pulsed PADRE as an effective vaccine in a mice TUBO model of breast cancer. J. Control. Release 303, 223–236. 10.1016/j.jconrel.2019.04.019 30999007

[B174] Zamani P. Navashenaq J. G. Teymouri M. Karimi M. Mashreghi M. Jaafari M. R. (2020). Combination therapy with liposomal doxorubicin and liposomal vaccine containing E75, an HER-2/neu-derived peptide, reduces myeloid-derived suppressor cells and improved tumor therapy. Life Sci. 252, 117646. 10.1016/j.lfs.2020.117646 32272178

[B175] Zamani P. Teymouri M. Nikpoor A. R. Navashenaq J. G. Gholizadeh Z. Darban S. A. (2020). Nanoliposomal vaccine containing long multi-epitope peptide E75-AE36 pulsed PADRE-induced effective immune response in mice TUBO model of breast cancer. Eur. J. Cancer 129, 80–96. 10.1016/j.ejca.2020.01.010 32145473

[B176] Zhang C. Yuan W. Wu Y. Wan X. Gong Y. (2021). Co-delivery of EGFR and BRD4 siRNA by cell-penetrating peptides-modified redox-responsive complex in triple negative breast cancer cells. Life Sci. 266, 118886. 10.1016/j.lfs.2020.118886 33310044

[B177] Zhang L.-S. Yan L.-X. Gao S. Long H. Xi Z. Li L.-Y. (2020). Self-assembling peptide–etoposide nanofibers for overcoming multidrug resistance. Chem. Commun. 56 (97), 15321–15324. 10.1039/D0CC06387H 33205785

[B178] Zhang L.-x. Xie X.-x. Liu D.-q. Xu Z. P. Liu R.-t. (2018). Efficient co-delivery of neo-epitopes using dispersion-stable layered double hydroxide nanoparticles for enhanced melanoma immunotherapy. Biomaterials 174, 54–66. 10.1016/j.biomaterials.2018.05.015 29778982

[B179] Zhang M. Lu W. (2018). Enhanced glioma-targeting and stability of LGICP peptide coupled with stabilized peptide DA7R. Acta Pharm. Sin. B 8 (1), 106–115. 10.1016/j.apsb.2017.11.004 29872627 PMC5985625

[B180] Zhao H. Liu Q. S. Geng H. Tian Y. Cheng M. Jiang Y. H. (2016). Crosslinked aspartic acids as helix‐nucleating templates. Angew. Chem. 128 (39), 12088–12093. 10.1002/anie.201606833 27572954

[B181] Zhao J. Jiang X. (2018). The application of sulfur-containing peptides in drug discovery. Chin. Chem. Lett. 29 (7), 1079–1087. 10.1016/j.cclet.2018.05.026

[B182] Zhi X. Jiang Y. Xie L. Li Y. Fang C.-J. (2019). Gold nanorods functionalized with cathepsin B targeting peptide and doxorubicin for combinatorial therapy against multidrug resistance. ACS Appl. Bio Mater. 2 (12), 5697–5706. 10.1021/acsabm.9b00755 35021563

[B183] Zhou X. Li C. Shao Y. Chen C. Yang Z. Liu D. (2016). Reversibly tuning the mechanical properties of a DNA hydrogel by a DNA nanomotor. Chem. Commun. 52 (70), 10668–10671. 10.1039/C6CC04724F 27506763

[B184] Zhou Y. Zhang S. Chen Z. Bao Y. Chen A. T. Sheu W. C. (2020). Targeted delivery of secretory promelittin via novel poly (lactone-co-β-amino ester) nanoparticles for treatment of breast cancer brain metastases. Adv. Sci. 7 (5), 1901866. 10.1002/advs.201901866 PMC7055583 32154067

[B185] Zhu L. Zhao H. Zhou Z. Xia Y. Wang Z. Ran H. (2018). Peptide-functionalized phase-transformation nanoparticles for low intensity focused ultrasound-assisted tumor imaging and therapy. Nano Lett. 18 (3), 1831–1841. 10.1021/acs.nanolett.7b05087 29419305

[B186] Zorko M. Langel Ü. (2005). Cell-penetrating peptides: Mechanism and kinetics of cargo delivery. Adv. Drug Deliv. Rreviews 57 (4), 529–545. 10.1016/j.addr.2004.10.010 15722162

